# Trial protocol for the Building Resilience through Socio-Emotional Training (ReSET) programme: a cluster randomised controlled trial of a new transdiagnostic preventative intervention for adolescents

**DOI:** 10.1186/s13063-024-07931-2

**Published:** 2024-02-23

**Authors:** Essi Viding, Alex Lloyd, Roslyn Law, Peter Martin, Laura Lucas, Tom Chin-Han Wu, Nikolaus Steinbeis, Nick Midgley, René Veenstra, Jaime Smith, Lili Ly, Geoffrey Bird, Jennifer Murphy, David Plans, Marcus Munafo, Ian Penton-Voak, Jessica Deighton, Kathleen Richards, Mya Richards, Pasco Fearon

**Affiliations:** 1 Clinical, Educational and Health Psychology, Psychology and Language Sciences, University College London, 26 Bedford Way, London, WC1H 0AP UK; 2 Anna Freud National Centre for Children and Families, London, UK; 3 Applied Health Research Institute of Epidemiology & Health, University College London, London, UK; 4 Department of Sociology, University of Groningen, Groningen, Germany; 5 Department of Experimental Psychology, University of Oxford, Oxford, UK; 6 Social, Genetic and Developmental Psychiatry Centre, Institute of Psychiatry, Psychology & Neuroscience, King’s College London, London, UK; 7 Department of Psychology, Royal Holloway, University of London, London, UK; 8 MRC Integrative Epidemiology Unit at the University of Bristol, Bristol Medical School, University of Bristol, Bristol, UK; 9 School of Psychological Science, University of Bristol, Bristol, UK; 10 London, UK; 11 Centre for Family Research, Department of Psychology, University of Cambridge, Downing Pl, Cambridge, CB2 3EB UK

**Keywords:** Adolescence, Mental health, Wellbeing, Transdiagnostic, Preventative, Indicated, Emotion, Social relationships, Interpersonal

## Abstract

**Background:**

Adolescence is a period of heightened vulnerability to developing mental health problems, and rates of mental health disorder in this age group have increased in the last decade. Preventing mental health problems developing before they become entrenched, particularly in adolescents who are at high risk, is an important research and clinical target. Here, we report the protocol for the trial of the ‘Building Resilience through Socioemotional Training’ (ReSET) intervention. ReSET is a new, preventative intervention that incorporates individual-based emotional training techniques and group-based social and communication skills training. We take a transdiagnostic approach, focusing on emotion processing and social mechanisms implicated in the onset and maintenance of various forms of psychopathology.

**Methods:**

A cluster randomised allocation design is adopted with randomisation at the school year level. Five-hundred and forty adolescents (aged 12–14) will be randomised to either receive the intervention or not (passive control). The intervention is comprised of weekly sessions over an 8-week period, supplemented by two individual sessions. The primary outcomes, psychopathology symptoms and mental wellbeing, will be assessed pre- and post-intervention, and at a 1-year follow-up. Secondary outcomes are task-based assessments of emotion processing, social network data based on peer nominations, and subjective ratings of social relationships. These measures will be taken at baseline, post-intervention and 1-year follow-up. A subgroup of participants and stakeholders will be invited to take part in focus groups to assess the acceptability of the intervention.

**Discussion:**

This project adopts a theory-based approach to the development of a new intervention designed to target the close connections between young people’s emotions and their interpersonal relationships. By embedding the intervention within a school setting and using a cluster-randomised design, we aim to develop and test a feasible, scalable intervention to prevent the onset of psychopathology in adolescence.

**Trial registration:**

ISRCTN88585916. Trial registration date: 20/04/2023.

**Supplementary Information:**

The online version contains supplementary material available at 10.1186/s13063-024-07931-2.

## Background

Existing research into potential mechanisms underlying mental health problems has typically had a narrow focus, usually examining a limited number of key processes and outcomes within a single disorder [1]. However, approaches focusing on a single process or outcome do not reflect the complexity and interconnectedness of the processes contributing to mental health difficulties in adolescence. Parallel to this, a significant limitation of past treatment and prevention work has been the focus on disorder-specific interventions. Yet, compelling evidence indicates that (a) the structure of mental health symptoms does not straightforwardly map onto traditional diagnostic categories; (b) comorbidity is high; and (c) individuals may experience multiple shifts from one diagnosis to another over time [2]. In that context, the *prevention* of adolescent mental health problems is a particularly complex challenge because it is often not possible to predict in advance which disorder(s) should be targeted and indeed young people may be at risk for more than one. In recognition of these challenges, attention is shifting to the development of transdiagnostic approaches to treatment and prevention [2]. The transdiagnostic approach aims to target common cross-disorder mechanisms, rather than specific diagnoses. This approach is consistent with the recent evidence that vulnerability to mental health difficulties can be represented by a General Psychopathology dimension (the ‘p-factor’), which emphasises the action of cross-disorder vulnerability mechanisms that predispose to overall poor mental health [3].

There is considerable evidence that *emotional processing* and *social relationships* are fundamental—and closely interlinked—transdiagnostic mechanisms underpinning resilience and vulnerability to mental health problems in adolescence [2–5]. Emotion processing and regulation mechanisms implicated in poor mental health continue to develop during adolescence [6]. Adolescence also represents a major developmental shift during which young people are exposed to changing social environments that must be navigated with increasing independence from family. Peers become more, and parents become less, significant [7]. Succeeding at navigating these new social challenges is crucial for ongoing mental health and wellbeing [7]. Prior work has established an association between emotion processing and mental health/wellbeing, and between social relationships and mental health/wellbeing [8]. Yet, little research has systematically investigated how adolescents’ emotion processing might actively shape their social relationships with peers or how social relationships with peers shape adolescent emotion processing in ways that either predispose to or protect against mental health problems.

In the proposed intervention, we focus on emotion processes (specifically negative emotion perception and emotion regulation) and social relationships as promising transdiagnostic targets for a preventative, indicated intervention. Further, we focus on schools with a higher-than-average incidence of poverty, in recognition of the increased risk for mental health problems in economically deprived populations [9]. Such interventions are particularly timely in the context of recent large-scale trials that have failed to find evidence to support the effectiveness of universal interventions [10, 11]. This paper details the protocol of the randomised controlled trial to test the efficacy of the ‘Resilience through socioemotional training’ (ReSET) intervention at reducing psychopathology in adolescents.

### Cognitive mechanisms

Targeted training can be applied to improve specific transdiagnostic emotional or cognitive processes under tightly controlled experimental conditions. There is good evidence that such training can alter emotional processes (including those selected for this intervention) and may positively influence mental health, in line with the presumed causal role of these mechanisms (e.g. [12]). In this study, we focus on training key cognitive-emotional processes that have been associated with the development of psychopathology in childhood and adolescence.

Sensitivity to perceiving negative emotions, especially sadness and anger, has been linked to several mental health conditions, including depression, anxiety and disruptive behaviour disorders [13–15]. Negative emotion perception is commonly measured by assessing sensitivity in perceiving sadness and anger [16]. Lower thresholds for perceiving negative affect are thought to impact adolescents’ own emotional state, as well as the formation and maintenance of their social relationships [17]. Indeed, a biased perception to perceive negative affect in others can lead adolescents to choose hostile responses, which in turn provoke negative responses from other individuals, thereby reinforcing biased emotion perception in a ‘vicious cycle’ [14, 15, 18]. However, previous research has demonstrated that negative emotion perception is amenable to intervention, interrupting the cycle of negative reinforcement between biased emotion perception and hostile patterns of responding. For example, training to increase adolescents’ threshold for perceiving negative affect has been found to improve symptoms of depression [19].

A further emotion process implicated in mental health conditions is emotion regulation, which refers to implicit and explicit processes and strategies involved in regulating emotional states (i.e. suppression, distraction, reappraisal; [20]). Adolescence is a sensitive period for emotion regulation development, with many individuals improving their use of adaptive emotion regulation strategies during this period [21, 22]. However, individual differences in the development of emotion regulation abilities are associated with risk for psychopathology in adolescence [23] and indeed poor emotion regulation skills in earlier childhood are associated with enduring mental health across childhood and adolescence [24]. Adequate downregulation of negative emotional states during childhood and adolescence plays a critical role in adaptive development and well-being [25] as in the quality of social relationships [26]. Importantly, emotion regulation difficulties and maladaptive regulation strategies have been associated with risk for psychopathology in children and adolescents [27], in particular internalising symptoms [28], anxiety disorders and disruptive behaviour disorders [13]. Consistent with the view that emotion regulation is implicated in the onset and maintenance of psychopathology, training that increases the use of adaptive regulation strategies (e.g. reappraisal and temporal distancing) can decrease symptoms of anxiety in adolescents [29]. However, previous interventions (e.g. [29]) have consisted of only a single session and improvements have not been sustained over longer periods of time in these studies. Interventions that have observed long-term effects of training emotion regulation typically utilise several sessions (e.g. [12]). To date there is tentative evidence that adolescents’ use of adaptive emotion regulation strategies can be increased, though it may be necessary to train these abilities over a number of sessions to see lasting benefits. This is the approach we take in the present intervention.

A considerable body of research has highlighted the links between interoception, the perception of the body’s internal state, and the ability to perceive and regulate emotions [30]. Further, interoceptive ability has been associated with several mental health outcomes, including anxiety, depression, alcohol and substance abuse [31–33]. It has been suggested that interoception may represent a common, transdiagnostic, vulnerability factor for psychopathology (i.e. that individual differences in interoception may contribute to ‘p-factor’ development; [34]). Recent theoretical work has also suggested that the rise in psychopathology observed in adolescence may be, in part, attributable to interoceptive impairments during this period [31, 34]. To examine the mediating role of interoception in the intervention outcomes, we measure interoceptive attention and accuracy before and after the intervention.

### Social relationships in adolescence

Social relationships, particularly peer relationships, are vitally important for mental health and wellbeing during this developmental period [35]. Recent meta-analytic work has documented robust associations between the number of peer friendships adolescents have, as well as their perceptions of these relationships, and mental health [36, 37]. For example, young adolescents with fewer friendships, or those with poorer-quality friendships, present greater internalising and externalising symptoms than those with more friends [36, 37]. Additionally, rejection and exclusion from the peer group engender loneliness and diminished self-esteem and increase risk for anxiety and depression [38–40]. Friendships are also more likely to break down when an adolescent experiences depression [41]. These studies suggest that peer friendships have a bi-directional relationship with mental health during adolescence.

While adolescence is a period in which the individual spends increasing time with peers and less time with parental figures (e.g. mothers, fathers, step-mothers, step-fathers or foster parents; [42]), the relationships between adolescents and parental figures are nonetheless important for mental health outcomes. Insecure parental attachment predicts conduct problems and emotional difficulties during adolescence [43], suggesting that parental attachment is a transdiagnostic risk factor for psychopathology. Further, positive relationships with caregivers are associated with positive mental health outcomes and wellbeing [44]. Indeed, positive relationships with parents and caregivers can also act as a protective factor from other social stressors, such as peer victimisation [45]. As such, a hybrid intervention focussing on improving the quality of social relationships along with training emotion processing skills implicated in mental health disorders could present an effective approach to the prevention of psychopathology in adolescence.

Inter-Personal Therapy (IPT) is an established evidence-based therapy that specifically focuses on improving interpersonal functioning and social relationships and was originally developed to treat depression [46]. This treatment has been adapted as a group intervention for adolescents (IPT-AST), which focuses on interpersonal problems young people may be experiencing, supports adolescents to recognise their feelings and link these to interpersonal conflicts in their relationships, and promotes interpersonal connections, social problem solving and communication skills [47]. Individual and group IPT-AST have good evidence of efficacy for depression in young people [48] and crucially there is increasing evidence for its effectiveness for a broad range of mental health outcomes [49, 50].

### Objectives

Addressing the cyclical relationship between relationships, emotion processing and mental health presents one potentially fruitful way to prevent the onset of psychopathology in adolescence. There is considerable evidence that intra-individual mechanisms related to emotional processing and inter-individual mechanisms linked to social relationships are both crucial for understanding risk for mental health problems in adolescence. However, little work has bridged the disciplinary boundaries between these approaches. Similarly, while interventions have been developed either to alter emotional processing mechanisms or to improve social relationships, these two approaches to intervention have been developed in almost complete isolation from one another and often fail to incorporate the views of those at whom the intervention is aimed. By working with clinicians, cognitive neuroscientists and young people themselves we aim to develop a novel prevention programme that specifically focuses on the close connections between emotional processing and social relationships. The integration of these two approaches to intervention could yield great scientific and clinical benefits and provide a much-needed new approach to mental health prevention for young people.

The aim of the current study is to test the impact of a novel hybrid prevention programme for young people, targeting key cognitive-emotional and social mechanisms. Specifically, we will test whether this novel hybrid intervention leads to better mental health and well-being outcomes in at-risk adolescents (highest 25% of general psychopathology risk), compared to those in a non-intervention arm. Further to this aim, we will examine whether the intervention’s impact on mental health and wellbeing is mediated by changes in emotional processing and social relationships. Our key hypotheses are as follows:

- (1) Our novel hybrid programme will lead to better mental health and well-being outcomes in at-risk adolescents (highest 25% of psychopathology risk), compared to those in the non-intervention arm.
- (2) The impact of the hybrid intervention will be mediated, in part, by changes to emotion processing and social relationships; specifically, the impact of the intervention will be mediated by:
  - A shift in emotion perception to view fewer faces as hostile.
  - A better ability to regulate emotions when presented with negative stimuli.
  - Improved interoceptive ability.
  - Improved peer acceptance and attachment with friends and parents, as well as decreased peer victimisation.

### Trial design

The ReSET intervention is a cluster randomised, controlled, multicentre superiority trial with (1:1) randomisation. The randomisation schedule will happen at the school year group level, meaning in each school we will have a younger and older cohort. As our study period will span two academic years, some baseline data collection and some intervention groups will take place in the following school year. In each school, one cohort is allocated to the hybrid intervention arm and the other to the non-intervention control arm; this is balanced across participating schools using block randomisation. Researchers are blind to participants’ group allocation during data collection. The intervention consists of eight group sessions, as well as one pre-group and one mid-group session that is completed individually or with the attendance of a parent or carer. All group sessions are 90 min in length, while individual sessions are 60 min each.

## Methods

### Study setting

Participants will be recruited from mainstream, state secondary schools in the South East of England. Intervention sessions will take place within the participants’ schools in a classroom designated by the host school.

### Eligibility criteria

Eligible participants will include pupils who are initially in years 7–9 (ages 11–14) at the time of the first baseline assessment, with each school running the study across 2-year groups only (i.e. either years 7 and 8, or years 8 and 9). We will seek consent from parents or carers, and the participant before they are enrolled in the study. Young people at *elevated risk* of mental health problems will be invited to participate, where *elevated risk* is operationalised as having a self-reported SDQ Total Difficulties score of 15 or higher in our screening assessment. This corresponds approximately to selecting the top 25% of this age group in the UK (see ‘Outcomes,’ below for a description of this measure). This estimate is based on self-reported SDQ scores from 2100 UK adolescents aged 10–15 derived from the Understanding Society panel survey (see [51]). The 75th percentile of the SDQ Total Difficulties Score in this sample was 15. Students will be considered ineligible if they have high rates of school absence or are identified by the school as being inappropriate for the group intervention due to a high risk of harm to self or others. We will not exclude participants on the basis of neurodivergence, such as Autism and ADHD, though pre-existing diagnoses will be recorded during the study, as will participants’ use of other mental health services.

Schools will be eligible to participate if more than 30% of their pupils qualify for free school meals (Department for Education, 2022). We chose this criterion to ensure socioeconomic diversity in our sample. A record of study sites can be found at: https://www.isrctn.com/ISRCTN88585916.

Facilitators will be mental health professionals either working for schools (e.g. school counsellors) or mental health professionals that provide services within schools from external agencies (e.g. NHS Educational Mental Health Practitioners). All facilitators will attend a 2-day training course (delivered by the clinical lead, RL, and a member of the research staff, AL) to provide them with information about the intervention, opportunities to roleplay group sessions, and training on how to administer the cognitive training tasks. Facilitators will have weekly supervision with the clinical lead, RL, throughout the duration of the intervention.

### Interventions

Our primary intervention will be a novel preventive intervention that combines psychoeducation about relationships and communication strategies, drawing on methods used in Interpersonal Therapy – Adolescent Skills Training (IPT-AST; [47]) and other comparable programmes (e.g. [46, 49]), with cognitive-emotional training that targets key emotion processing abilities implicated in the onset and maintenance of psychopathology [5, 15, 34]. We first describe the structure of the new group intervention which integrates the focus on communication with the battery of the cognitive-emotional training tasks. We then describe the structure of each of the training tasks in greater detail.

#### The ReSET group intervention

ReSET is a manualised intervention (including cognitive-emotional training elements) delivered in a group-based setting with up to 10 adolescents allocated to each group. Sessions will be held weekly over an 8-week period and each session lasts between 90 and 120 min. The sessions will focus on developing adolescents’ understanding of the links between their social relationships, emotions, and mental health. Here, we describe the structure of our intervention that integrates the cognitive-emotional training tasks with psychoeducation about communication and mental health.

The intervention will begin with young people having an individual meeting with the group facilitator to discuss the aims of the groups, their goals for taking part, and any questions they have about the sessions. These individual meetings will be used to identify where the young person may be experiencing interpersonal difficulties that can be addressed in the group sessions. Issues arising from interpersonal difficulties will be set as explicit goals by the young person (with the aid of the facilitator), which will allow the young people to self-monitor their progress throughout the duration of the intervention.

Each of the group sessions will begin with participants completing the Child Outcome Rating Scale (CORS), a clinical measure designed to monitor an individual’s progress during the therapeutic process [52, 53]. The CORS measures several domains of the young person’s life functioning, including their individual wellbeing, interpersonal wellbeing, social role, and overall wellbeing [53]. The CORS has been validated with adolescents aged 11–15 [52]. Clinical cut-offs will be used by facilitators to identify young people who may need additional support during the group, or those that may benefit from more targeted services. The CORS will also be utilised during the intervention sessions to consider links between the strategies discussed in the sessions, their impact on how participants communicate with those around them, and the relationships between social interactions and wellbeing. Drawing links between social interactions and wellbeing will be a core component of the psychoeducation content of the intervention, particularly in relation to communication.

The main body of the group sessions will include a mixture of discussion activities, role-plays, and reflective exercises. These sessions draw on principles from IPT and IPT-AST and integrate these with consideration of the role that emotion processing plays in one’s social interactions. The sessions will aim to teach participants about effective communication and emotion processing strategies that can improve social relationships and subsequently mental health. Sessions will end with participants being provided with homework to complete before the next session. The eight sessions will be structured to gradually introduce participants to the psychoeducation content and training tasks in early sessions before they are encouraged to apply the skills developed to their personal experiences.

**Fig. 1:**
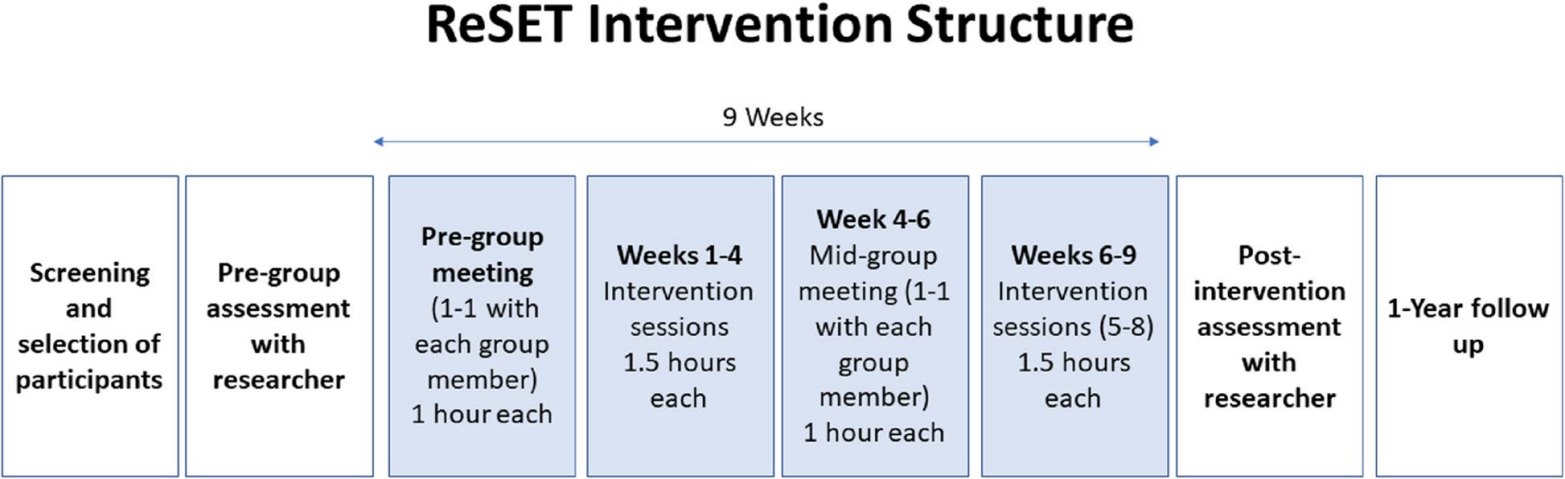
Structure of the hybrid group intervention and research assessments

The initial phase of the intervention will take place during sessions 1–4 (see Fig. 1). During these sessions, participants will be introduced to the cognitive-emotional training tasks and instructed how to complete them. Participants will also be introduced to the core concepts of the intervention; namely, the links between social relationships, emotion processing and wellbeing. Participants will be guided to explore the impact their emotions and responses can have on those around them, which will be used to identify opportunities to utilise adaptive communication or emotion processing skills. In sessions 2–4, participants will be provided with specific communication and emotion processing strategies designed to improve interpersonal interactions. Strategies will be introduced that either have an intra-personal focus (e.g. breathing to calm themselves down before responding) which are referred to as ‘Me Strategies’, or an interpersonal focus (e.g. picking the right time to have a conversation with another individual) which are referred to as ‘We Strategies’. Using role-plays of fictional scenarios, participants will identify moments where features of an interaction led to a dispute or difficulty and are encouraged to consider alternative ways to approach the scenario using the Me and We Strategies. The fictional scenarios that will be used in the current intervention were co-produced with a separate group of young people who did not take part in the intervention.

In each session, participants will complete an emotion regulation training task and in sessions 2–7 participants complete an emotion perception training task. Each of these tasks is delivered via a tablet that is provided to schools by the research team. From session 4 onwards, participants also complete breathing exercises designed to train the ability to identify one’s internal bodily signals, which is aimed at improving participants’ interoceptive abilities [54]. The intervention will include specific psychoeducational content on the features of emotion processing that the cognitive-emotional training tasks target, so that young people understand how the training relates directly to the ‘Me’ and ‘We’ strategies discussed in the group. In sessions 2–4, the group leader will facilitate activities to discuss how the cognitive-emotional mechanisms trained using the tasks (e.g. emotion perception) relate to our daily experiences, including interactions with those around us. The group facilitator will encourage participants to identify opportunities to use the skills developed through the cognitive-emotional training battery to improve their interpersonal interactions.

The middle sessions of the intervention (sessions 5 and 6) are designed to encourage participants to actively apply the ‘Me’ and ‘We’ Strategies to scenarios relevant to their lives. The scenarios that the young people discuss are intended to be relevant to the goals they identified in the individual meetings prior to the group sessions to support them to achieve these goals. The group facilitator will guide participants to reflect on conversations the participant has had and plan future interactions using the strategies outlined in sessions 1–4. The group facilitator will encourage participants to consider how different strategies can be used in combination, with the aim of providing participants with a ‘toolkit’ of adaptive interpersonal and emotion processing strategies that improve their interactions with individuals in their lives. Participants will be tasked with homework to practice conversations at home and report how these interactions went in the following session.

The closing sessions (sessions 7 & 8) of the intervention are designed to prepare participants to use these strategies independently, rather than relying on prompts from the group sessions. In these closing sessions, the group leader will review which of the communication and emotion processing skills have been useful and will work with participants to identify methods that encourage the self-generated use of these strategies outside of the sessions. During these final sessions, the young people will be asked to rate their progress on the goals they identified prior to starting the group sessions as a reflective exercise.

#### Interpretation bias training

To index and train biases in emotion perception we will utilise a task that measures participants’ subjective perception of ambiguous facial expressions. The interpretation bias training task requires a forced choice judgement as to whether the presented face is displaying an ‘angry’ or ‘happy’ expression. We will deploy an existing training protocol that has been shown to successfully shift perception from angry towards happy emotions, and significantly improve symptom outcomes in adolescents [14, 19]. On each trial the participant will be presented with a face and asked to categorise whether the emotion presented was happy or angry. There are a total of 15 facial expressions that range from unambiguously happy to unambiguously angry, with images between the unambiguous extremes linearly morphed at equal intervals from the two unambiguous emotions [55]. Stimuli will be presented for 150 ms after which a mask will be presented for 250 ms.

During the training, participants will first complete an assessment task where they are asked to make forced-choice judgements about the stimuli without receiving feedback. The assessment phase consists of 45 trials, meaning that the full stimuli set of 15 images is presented three times. Participants’ rating on the assessment task is used to calculate a ‘balance point’, which is a measure of the point within the stimuli set at which participants shift to perceiving stimuli as displaying anger rather than happiness [14, 19]. The balance point is calculated as the number of faces categorised as happy, divided by three [56]. We will introduce an attention check to identify patterns of random responding, such that participants who respond incorrectly to five of the most extreme morphs will be asked to restart the assessment task, with a prompt reminding them to pay attention to the task.

**Fig. 2:**
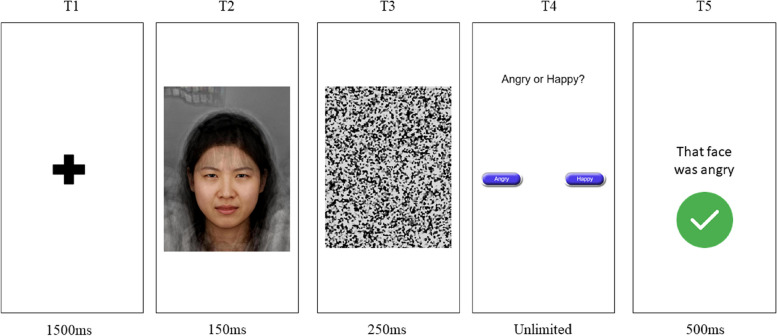
Negative emotion perception training task. At T1, participants are presented with a fixation cross, followed at T2 by an ambiguous facial stimulus. This facial stimulus is followed at T3 by a visual mask. At T4, participants are provided with unlimited time to make a categorical judgement about the emotion they perceived the face to be displaying. At T5, participants are provided with feedback, along with the ‘correct’ emotion. Note, the assessment task follows the same structure, but does not include the feedback screen (T5)

The training section of the task mirrors the structure of the assessment phase, with the addition of feedback determined using the participant’s balance point. Participants complete 90 trials in which they receive feedback that faces that are two images above their balance point along the linear continuum are categorised as ‘happy’. On training trials, participants will categorise stimuli as either happy or angry, after which they will be provided with feedback about whether they are correct, along with a visual cue indicating whether they are correct or incorrect (see Fig. 2). Feedback will be calibrated to participants’ balance point to shift their individual bias away from perceiving negative emotions. However, a threshold will be set such that the three most unambiguous faces will always be classed as the correct emotion (e.g. participants will always receive feedback that the three most angry faces are exhibiting anger). As such, there is a ceiling for the feedback participants can receive in these sessions.

The emotion perception training is completed six times over the course of the intervention (in sessions 2–7). The number of training sessions was determined based on previous research (e.g. [19]) as well as pilot data which suggested that training effects asymptote by the fifth to sixth training session. Across training sessions, different avatars will be used as stimuli in the task to ensure participants are trained using individuals from diverse ethnic backgrounds and the presentation order of these avatars is randomised across participants. For each participant, the same avatar is presented for the first and final training sessions. These avatars were drawn from stimuli used in previous research [57]. Altogether, the task takes approximately 10 min to complete.

#### Emotional regulation training

**Fig. 3:**
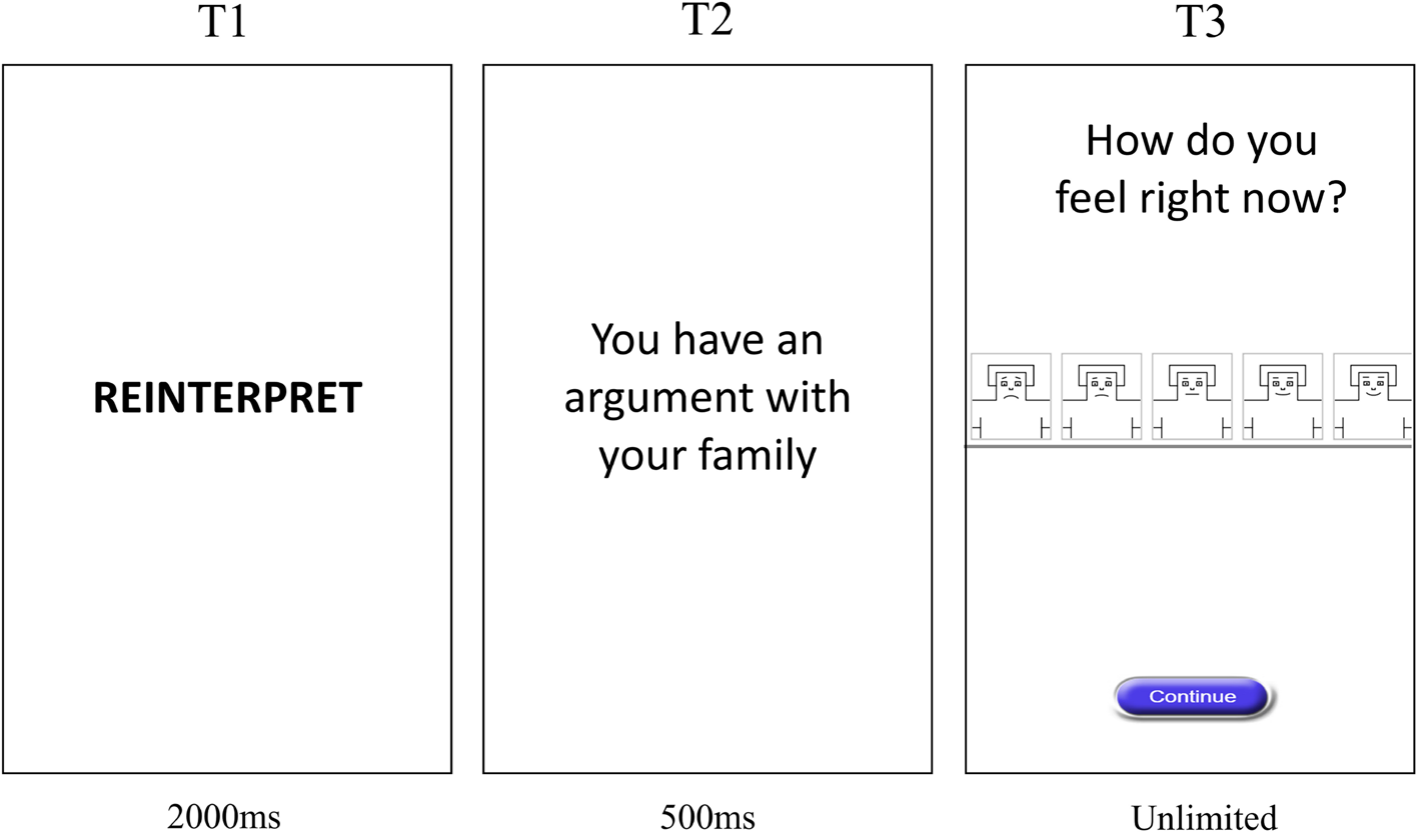
Schematic outline of the emotion regulation training task. At T1, participants are presented with an emotion regulation strategy (either ‘REINTERPRET’ or ‘DISTANCE’) before being presented with a scenario designed to elicit a negative emotion (T2). At T3, participants are asked to report their affect on a Self-Assessment Manikin

The emotion regulation training is derived from tasks widely used in the literature on affective cognitive control [58] and has been modified for adolescents [59]. The task aims to train adolescents to utilise adaptive emotion regulation strategies (i.e. reappraisal and distancing) when presented with negative scenarios. Before starting the task, participants will be presented with developmentally appropriate instructions informing them about the reappraise and distance emotion regulation strategies [60]. At the start of each block participants will be instructed to use an emotion regulation strategy (either ‘reappraise’ or ‘distance’) when viewing the stimuli. After receiving instruction about which strategy to use, participants will be presented with a written scenario detailing a negative social interaction (e.g. ‘your friend ignores your text’), which lasts for five seconds. This scenario is presented both visually and auditorily, consistent with previous research [61]. The presentation of the written scenario will be followed by self-report of the strength of negative affect (see Fig. 3).

The scenarios we present to participants will be selected from a mixture of those used in previous research [59] and from novel scenarios co-produced with adolescents for the purposes of this study. To develop these novel scenarios, we conducted a co-production session with 23 adolescents aged 16–17 who were asked to document negative interpersonal situations that would be relevant to adolescents aged 11–14. Following this initial session, we asked a separate group of 19 young people aged 16–17 to rate each of these scenarios using Self-Assessment Manikins (SAM; [62]). The SAM provides a pictorial representation of how the participant is feeling on different domains of affect. We asked this group of young people to rate whether the scenario made them feel unhappy (ranging from 1–5 where 1 was happy and 5 was unhappy), excited (ranging from 1–5 where 1 was excited and 5 was calm) and in control as opposed by being controlled by external forces (ranging from 1–5 where 1 was in control and 5 was controlled). As we only aimed to train young people to regulate negative emotions, we excluded any scenarios that were rated below three on the SAM rating for happiness, as this would indicate the scenario produced positive emotions.

The emotion regulation task will be split into four blocks each containing eight scenarios (32 trials in total). Each block instructs participants to use one of the emotion regulation strategies (‘reinterpret’ or ‘distance’) and are presented in alternate sequences (i.e. a reinterpret block is followed by a distance block, which is followed by a reinterpret block). The order with which the blocks are presented will be counterbalanced across sessions. We will structure the emotion regulation task such that early trials presented scenarios that were only mildly negative (as rated by the adolescent focus group) and increased in the strength of negative affect as the task progressed. At three intervals during the task, we will ask participants ‘how engaged are you feeling?’ as a measure of attention. The task takes approximately 12 min to complete.

#### Adherence

Adherence to the intervention is promoted using verbal encouragement from school contacts who will liaise between the facilitation team and study participants. School liaisons also communicate with class teachers to ensure students are released from their usual classes to attend sessions. Further, the mid-group meeting is used as an opportunity to identify any barriers to participation and, should any barriers be identified, provide additional support to increase adherence. Participants’ adherence to the researcher assessments is encouraged by remunerating participants for these sessions.

Throughout the intervention, adherence is monitored by recording attendance as well as monitoring the cognitive training data collected in each group session. This monitoring protocol includes attention measures collected during the cognitive training tasks. In the emotion regulation training task, participants are asked at three timepoints to report how engaged they were feeling to indicate their adherence to the training. For the interpretation bias training, participants are asked to repeat the first 45 trials measuring their balance point if they incorrectly identify the emotion in more than three of the extreme morphs. For example, if a participant incorrectly identifies the extreme image expressing anger as ‘happy’ more than three times, they are asked to repeat the assessment phase. We will report data on participants who are asked to repeat the assessment phase as a measure of adherence to the interpretation bias training.

Finally, at three timepoints during the intervention (group sessions 1, 2 and 5) a member of the research team conducts an audit of the group session. This process involves a member of the research team attending the session to monitor fidelity to the intervention manual, assessing whether key concepts are covered and explained using terminology described in the manual using an adherence checklist. The audits are also used to identify interpersonal dynamics within the group that may affect adherence to the intervention (e.g. challenging behaviour).

#### Intervention development

We conducted a pilot and feasibility study of the intervention prior to the main trial. During this development process, we piloted and feasibility-tested an interoception training protocol for adolescents. However, this training was not well received by adolescents in our pilot and feasibility studies, as participants did not engage with the task. Due to the low acceptability of the interoception training, this component of the cognitive training was removed from the final intervention. A paper detailing the intervention development process in full is in preparation.

## Outcomes

### Primary impact outcomes: general psychopathology and wellbeing

The two primary outcomes are psychopathology symptoms and mental wellbeing. Psychopathology symptoms will be assessed using the Total Difficulties Score of the Strengths and Difficulties Questionnaire (SDQ; [63]). Mental Wellbeing will be measured by the summary score of the Warwick and Edinburgh Mental Wellbeing Scale (WEMWBS; [64]). The primary endpoint will be the immediate post-intervention assessment point. Data for the primary impact outcomes will be collected prior to start of the intervention group, immediately after the intervention group has been completed, and 12-months from the first data collection timepoint (i.e. the data collected prior to the start of the intervention group). Note, these data collection timepoints are for participants allocated to both the intervention and control groups.

### Secondary impact outcomes

We will also include General Psychopathology, internalising problems and externalising problems as secondary outcomes based on a confirmatory factor analysis of the items from the Emotional Symptoms and Conduct Problems scales in the Strengths and Difficulties Questionnaire (SDQ; [63]) and 15 items from the Me and My Feelings measure (M&MF; [65]). The factor analysis will estimate a bifactor model as presented in Patalay et al. [66]. This model yields three factors: a general psychopathology factor (‘p-factor’), a specific factor measuring Internalising Problems, and another factor measuring Externalising Problems. We will assess the fit of this model to our data and estimate an amended model if necessary for estimation convergence or goodness of fit.

Additional secondary mental health outcomes will be obtained using the Patient Health Questionnaire (PHQ-8; [67]), and Generalised Anxiety Disorder Assessment-7 (GAD-7; [68]). The PHQ-8 and GAD-7 will be included as part of the Wellcome Trust/NIH common metrics for mental health [69]. Further, we will collect self-report measures of substance use: specifically, alcohol and drug use disorders using the Alcohol Use Disorders Identification Tests (AUDIT) and Drug Use Disorders Identification Tests (DUDIT). For the PHQ-8 and GAD-7, we will use established cut-off scores to establish caseness [70].

Sleep phenotypes relevant to mental health will be measured using a bespoke questionnaire comprised of items from the Pittsburgh Sleep Quality Index [71], several novel items designed to assess insomnia in a developmental sample (see Additional file 1: Appendix), and an item used to assess diurnal preference [72]. These items were selected based on their relevance to mental health outcomes and included items that asked participants to rate their typical bedtime and waketime, how many hours they typically slept for, and self-reported difficulties sleeping. The full set of items are reported in the Additional file 1: Appendix.

For intervention and control participants, data for the secondary impact outcomes will be collected prior to start of the intervention group, immediately after the intervention group has been completed, and 12-months from the first data collection timepoint.

### Mediating mechanisms

#### Emotion perception

To assess biases in emotion perception pre- and post-intervention, we will use two well validated tasks and one validated questionnaire. Biases in emotion perception will be assessed using the Interpretation Bias Assessment Task (see Interpretation Bias Training). However, for the purposes of assessing the efficacy of our intervention, we will not include trials where participants receive feedback about their performance. Participants’ balance point will be used as a metric of their bias to perceive negative emotions. Each participant will be presented with the same face when assessed pre- and post-intervention [57], but the avatar they are presented with will be randomised across participants. There is evidence that previous exposure to faces does not change emotion perception ratings on this task [14].

Emotion perception bias will also be assessed using the emotional intensity morphing task [73]. In this task, participants observe a face that gradually morphs to display an emotion (either sadness, anger, fear or happiness) or begins by displaying the emotion and gradually morphing to display a neutral emotion [74]. On trials where the face morphs to display an emotion, participants are asked to press the screen when they perceive the emotion, whereas on trials where the face morphs to exhibit a neutral emotion, participants are asked to press the screen when they no longer perceive the emotion. Self-report data of emotion perception biases will be collected using the attributional styles questionnaire [75].

#### Emotion regulation

Emotion regulation will be measured using two emotion reappraisal tasks and the emotion regulation scale. The first emotion regulation task utilises a set of scenarios (different from those use in the emotion regulation training). Participants will be presented with standardised instructions to either look at the scenario and attend to their emotions or reduce their negative affect towards the scenario. There are 24 assessment trials in total split equally between look trials and regulate trials. Self-reported affect recorded after each scenario (using the self-assessment manikin; [62]) will be used to assess emotion regulation success. After completing the assessment task, participants will be provided with one block (four trials) of positively valenced scenarios and asked to attend to the emotions elicited by the scenario. This final, positively valenced block was introduced to avoid inducing persistent negative affect in participants.

In the second emotion regulation task, participants are asked to regulate their emotions in response to images, rather than written scenarios. Images are drawn from the IAPS picture set [76], and have been selected based on those previously been used with developmental populations [77–79]. Participants will be asked to either look at the images and attend to the emotion the image elicits, or reduce the affect they feel in response to the image. This task comprises of four blocks each containing five trials. Two blocks will instruct participants to look at the image and two blocks will instruct participants to reduce their negative affect. After completing the task, participants will be provided with a single block of positive images (5 trials) to avoid inducing persistent negative affect in participants.

Self-report emotion regulation will be assessed using the Emotion Regulation Questionnaire for Children and Adolescents (ERQ-CA; [80]), which is a 10-item scale measuring use of emotion regulation strategies separated into positive emotion regulation strategies (i.e. cognitive reappraisal) and negative emotion regulation strategies (i.e. suppression). For each of the two emotion regulation strategies, scores are summed, with higher values denoting greater use of that emotion regulation strategy.

#### Interoceptive accuracy and attention

Interoception ability will be measured using the Phase Adjustment Task (PAT; [81]), the interoceptive accuracy scale [82] and the interoception attention scale [83]. The PAT involves detecting heartbeats with the device camera and flash. Heartbeat information is used to present tones that are asynchronous with the participant’s heartbeats. Without feeling their pulse, participants must judge synchronicity by adjusting the phase relationship between tones and heartbeats such that tones become synchronous with heartbeats. The starting phase that determines the delay between the tone and participants’ heart beats is randomised across trials [81]. The task begins with 10 ‘screener’ trials in which participants match a tone representing their heartbeat to an asynchronous tone. After completing these practice trials, participants complete 20 assessment trials in which they must match a single tone to their heart rate. To assess performance on the PAT, we will calculate the consistency of selected phase relationships or ‘delays’ across trials [81]. Fifteen trials are required as a minimum to calculate performance on this task. Greater consistency indicates greater interoceptive accuracy whereas more inconsistent responding indicates poorer interoceptive accuracy. Participants with greater consistency are categorised as ‘interoceptive’ whereas participants who are inconsistent are categorised as ‘non-interoceptive’ [81]. In addition to these data, the PAT also records engagement data (e.g. time spent on trials).

Self-report measures of interoception will be collected using the interoceptive awareness and interoceptive attention scales. The interoceptive attention scale measures the extent to which the individual attends to internal signals [83], while the interoceptive accuracy scale measures how adept individuals are at recognising internal bodily signals [82]. Both measures are 21-items, and scores are summed with higher values denoting greater self-reported accuracy or attention (ranging from 21–105).

#### Self-perception

Three questions from the Social Network Analysis of Risky Behaviors in Early Adolescence (SNARE; [84]) will be used to assess the participants’ perception of themselves. The questions are introduced with the stem ‘If you compare yourself with most of your classmates…,’ and participants rate themselves on attributes ‘…how nice are you?,’ ‘…how popular are you?,’ and ‘…how mature are you?’ on a 5-point scale ranging from 1 (‘Much less’) to 5 (‘Much more’).

#### Self-ratings of social relationships

To assess participants’ relationships with those in their social networks, we will measure parent and peer attachments, bullying victimisation and loneliness. Parent and peer attachment will be measured using the inventory of parent and peer attachment (IPPA; [85]). This measure is split into questions regarding relationships with the participant’s mother figure, father figure and close friends. For primary female and male caregivers, there are 28 items assessing parental attachment and for close friends there are 25 items. For each relationship, the scale measures several subdomains including trust, communication and alienation, with higher values denoting more positive attachments. Bullying victimisation will be measured using the multidimensional peer victimisation scale (MPVS; [86]). The scale comprises of 21 items scored on a 5-point response scale. Scores on this scale are summed, with higher values indicating greater victimisation. Finally, loneliness will be measured using the UCLA loneliness scale (version 3; [87]), a 20-item measure of the participant’s isolation from others with higher values denoting greater loneliness.

#### Peer social networks

Network nomination will be used to assess the participants’ friendship networks. Participants will be asked to nominate [88, 89] (i) Who in your year are your best friends? (ii) Who is in your friendship group? (iii) Who are the most popular kids in your year? (iv) Which kids in your year group do you like? Participants can nominate an unlimited number of same-sex and other-sex pupils as they wish for these questions. Two additional questions are also included to assess the effects of intervention: (i) Who gives good advice to you when you are feeling upset? (ii) Who in your year makes others feel accepted/like they belong? In addition, students will also complete the Inclusion of Self Scale (IOS Scale; [90]). This scale asks participants to indicate how close they feel to their peers using Venn diagrams indicating increasingly overlapping circles. Greater overlap indicates the participant feels closer to the nominated peer.

Unlike the other secondary outcome measures, social network data will be collected alongside the screening data from entire year groups in addition to the pre- and post-assessment timepoints with study participants. To construct social networks, we require that the full year group nominate peers for each of the questions to situate study participants within these wider social networks and assess their relations to their peers. The social network data will be completed by year groups in the school term preceding the first group, in Summer 2024 after all groups have run across all study sites, and at a 12-month follow-up in Summer 2025.

### Control variables

#### Backwards digit span

The backwards digit span is a short task measuring working memory capacity [91] and was included as a control variable as it is a measure of general cognitive ability, which may affect the efficacy of the cognitive training [92]. Participants are presented with sequences of numbers and are asked to remember the sequence in reverse order. If participants successfully remember the sequence in reverse order, the number of digits increases by one on the next trial, whereas if participants fail to remember the sequence in reverse order, the number of digits decreases by one on the next trial. The version of the task we use is administered via a tablet as in previous research [93]. Backwards Digit Span data will be collected before the start of the intervention group, immediately after the intervention has been completed and 12 months from the first data collection timepoint for both intervention and control group participants.

### Process evaluation

#### Focus groups and interviews

On completion of the 8-week intervention, a subgroup of participants who took part in the study will be invited to take part in a focus group or 1–1 interviews to discuss their experiences of the group sessions. The focus group and interviews will be facilitated by an independent researcher (i.e. not the group facilitators). Interview and focus group schedules will be semi-structured and will encourage participants to draw on their experience across the full intervention. Participants will be asked about barriers to participation, strategies they found particularly helpful from the sessions, and whether the novel integration of the cognitive-emotional training tasks with psychoeducational content around communication was clear. Questions for the focus group and interviews were developed in collaboration with young people aged 16–17 (*N* = 23), who identified questions that would be important to assess participants’ experience of the intervention, which included features of the group composition and the burden of taking part in the intervention.

#### Stakeholder feedback

In addition to conducting focus groups with adolescents, all stakeholders (intervention group facilitators, head teachers, Special Educational Needs Coordinators and mental health leads) in each site will be invited to complete a survey at the end of the delivery phase. The purpose of this feedback will be to examine facilitators and barriers to delivery and to assess their views on scalability. In addition, we will hold interviews with a subsample of stakeholders to gather qualitative feedback about the delivery of the intervention.

#### Qualitative analysis

Interviews and focus groups with participants and stakeholders will be analysed using framework analysis [94]. This is a qualitative research method involving the research team developing an initial framework based on the core research questions. Each focus group transcript is then thematically analysed in that context to identify core themes. This approach allows the team to draw on both a priori issues (as determined by the interview schedule and topics already identified in the literature) and on themes that emerge inductively from the data.

## Assignment of interventions

### Timing of randomisation

Randomisation will be performed at each school once that school has been provided with a list of participants scoring within the top 25% (i.e. above the cut-off score of 15) of the sample from both year groups. All eligible students will be invited to participate, and students will be randomised in each year group in a numerical order: (a) 1–10 (Group 1)—Term 1, (b) 11–20 (Group 2)—Term 2, and (c) 21–30 (Group 3)—Term 3. As our intervention runs over two academic years in some schools (April 2023–March 2024), some cohorts will transition to the next academic year before the completion of the intervention. For example, the group that is in year 7 when the school is randomised will be in year 8 during the final intervention group run in that school (January–March 2024).

**Fig. 4:**
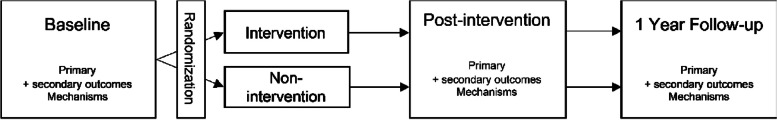
Schematic of the project timeline

A random selection of eligible adolescents will subsequently be invited to participate in the main study. Parents will receive consent forms detailing the aims of the study, the structure of the intervention and the time commitment expected of participants (see Additional file 1: Appendix), which will also include a consent form. Once parental consent has been obtained, participants will be randomly allocated to one of the three school terms (i.e. the sample will be split into thirds with one third taking part in term 1, a second third taking part in term 2, and the final third taking part in term 3). At the beginning of the allocated term, consent will be sought from the young person, and they will complete the assessment battery comprising of mental health, emotion processing and social relationship measures (see Fig. 4). Participants allocated to the intervention arm of the study will then complete 10 sessions inclusive of individual pre-group sessions, 8 weekly group sessions, and a mid-group session. Participants allocated to the control arm of the study will complete their usual classroom schedule.

Procedures are also in place to account for drop-out of participants. Should any young people drop out before the intervention has begun (prior to the one-to-one meeting with the group facilitator), the next consecutive student in the list will be invited to take their place (i.e. student 31, student 32, etc.) and baseline assessments will be conducted. On the other hand, any students who ‘drop-out’ between baseline assessment and intervention will be treated as ‘intention to treat’ and therefore will be invited to take part in the follow-up assessment even if they do not participate in the ReSET group.

For the integrity of the randomisation to be preserved, it is crucial that the allocation of young people to a treatment within a given school occurs *after* all eligible children from that school (and their parents) have been screened and consented to take part. If a child or a parent knew, at the point of baseline assessment and/or at the point of making the decision whether to take part, which treatment arm they would be signing up for, then participation would not be independent of randomisation. This would risk selection effects which have the potential to cause bias in the estimation of intervention effects. As such, we will screen the entirety of the participating year groups at the first screening timepoint, and students will only be informed which group they have been allocated to only after they have completed their baseline assessment. We expect modest increases in symptomology between the screening phase and the beginning of the third set of assessments in line with population-level changes to psychopathology during adolescence (e.g. [95]). However, it is vital to the randomisation procedure that participants and their parents consent to take part in the study *prior* to when randomisation takes place, which necessitates that we screen and consent participants in the top 25% of SDQ scores at a single timepoint. We will collect SDQ data at our baseline assessment in addition to this screening questionnaire, which will allow us to adjust for changes to general psychopathology between the first screening timepoint and when the participant completes the baseline assessment.

### Randomisation schedule

Randomisation will be carried out in randomly permuted blocks of size 2. Each block has two elements:

- Cohort1-INT: Year 7 cohort receives the intervention, Year 8 cohort is the control group
- Cohort2-INT: Year 7 cohort is the control group, Year 8 cohort receives the intervention

A statistical programme to generate a randomised list of permuted blocks will be written by the project statistician, PM, using the R Software for Statistical Computing. This programme will then be used by an independent statistician not involved in the study to generate a randomised list. The independent statistician will use a new seed number to generate the list, and pass this on to the trial manager, LL. The trial manager will use the list to allocate year groups to treatment arms, going down the list in the order in which schools are ready to be randomised (see Additional file 1: Appendix for R script).

### Blinding

Researchers conducting any assessments with participants will be blind to the allocation. Should any researchers become unblind to the allocation (e.g. due to a disclosure by a school staff member), this information will be recorded and reported with the trial outcomes. Only the independent statistician, researcher coordinator and PIs will have knowledge of the school allocation. The primary analysis will be conducted by the project statistician. A fully blinded analysis is not possible because of the partially clustered data structure (intervention participants are clustered within their groups, while control group members are not).

### Timeline

Schools will be recruited in the academic year prior to the start of the intervention (i.e. for schools participating in April 2023–July 2024, we will recruit schools from September 2022–September 2023). We intend for each participating school to run a minimum of two intervention groups across two school terms and a maximum of three intervention groups across three school terms. Once schools have been recruited, consent forms will be sent to parents/carers of the participating year group cohorts asking them to indicate if they would not like their child to complete the screening measures (i.e. we will utilise an opt-out procedure). Once the deadline for returning consent forms has passed, participating adolescents will complete the SDQ, Me and My Feelings Questionnaire, and Peer Social Network nominations. We will then invite students who report SDQ total difficulties scores of 15 or higher—corresponding to the top 25% nationally, as estimated based on recent national data [51].

**Fig. 5:**
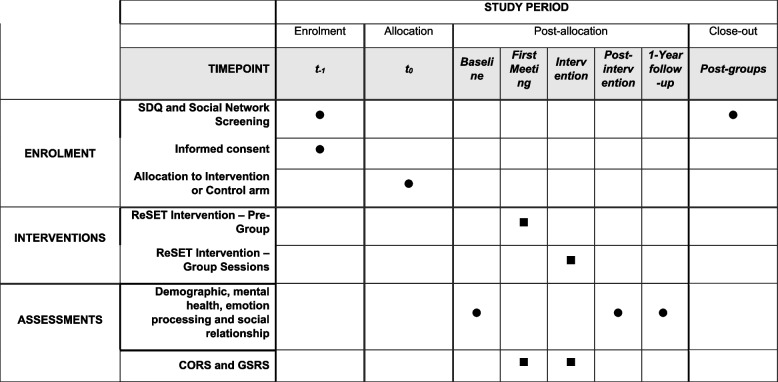
SPIRIT checklist outlining the timeline of the pilot intervention.⏺ = all participants are involved; ⏹ = only participants in the intervention condition are involved

After the conclusion of the intervention, participants from both the intervention and control arms of the study will complete the assessment tasks measuring mental health, emotion processing and social relationship detailed in the “Outcomes” section. Once all three groups have been run within a school, we will collect follow-up measures consisting of the SDQ, M&MF and peer nomination to allow us to estimate social networks within the year group. SDQ, M&MF and peer nomination data will be collected from all students in the year group (not just those eligible to take part in the groups). Finally, 1 year following the completion of the intervention, participants from the intervention and control arms of the study will complete the full battery of assessments tasks as well as the social network measures (see Fig. 5).

## Sample size

### Sample size calculation

The present study is a cluster-randomised trial. The primary analysis model to investigate Hypothesis 1 (effectiveness of the intervention compared to a passive control) is a partially clustered mixed effects model, which takes account of the fact that participants will be clustered within intervention groups for those in the intervention arm. The analysis model also takes account of clustering within schools and within school years (see below for details on the analysis model). Power analysis for the evaluation of the intervention was conducted using published formulae for partially clustered data [96]. The two primary outcomes are the SDQ Total Difficulties Score and the Short Warwick Edinburgh Mental Wellbeing Scale (SWEMWBS).

#### Preliminary considerations

There are no trials of preventive interventions similar to ours on which we could base hypotheses about the likely size of the effect of our intervention. A meta-analysis comparing the effect of psychotherapy relative to waitlist control estimated an average standardised effect of Hedges’ *g* = 0.75 [97, 98]. Given that our intervention targets an at-risk population, rather than a clinical one, we assumed that the minimum standardised effect size we wished to detect was about half Munder et al.’s [97] estimate. We thus used a standardised effect size of δ = 0.4 for the power analysis calculations, in conjunction with considerations about clinically meaningful effect sizes.

Psychopathology will be assessed using the SDQ Total Difficulties Score. There is no consensus regarding how many point difference on the SDQ constitutes clinically meaningful change [99]. We considered that a post-intervention difference between 1 and 2 points between the intervention and control groups would be clinically meaningful. A 1-point difference corresponds to one of 20 assessed symptoms improving by 1 point; a 2-point difference corresponds to one symptom improving by 2 points, or two symptoms by 1 point each. We estimated the standard deviation of the SDQ Total Difficulties Score using the data set from the Understanding Society study (*n* = 2100) that we also used to estimate our cut-off for eligibility (top 25% of SDQ scores). In the ‘eligible’ group (who had a score of 15 or higher, *n* = 604), the mean score was 19.1, with standard deviation 3.6. Raw effect sizes of 1, 1.5 and 2 points would correspond to standardised effect sizes of about 0.28, 0.42 and 0.56. We thus considered that, by powering for a standardised effect size of 0.4, we would be able to detect a 1.5-point difference in SDQ Total Difficulties Scores post-intervention.

*Mental Wellbeing* will be assessed by the WEMWBS. There are few published data sets allowing estimation of the distribution of this scale among younger teenagers, and none for our specific at-risk population (top 25% in mental health problems). As the closest available approximation, we used data from the 2018 sweep of the Millennium Cohort Study (MCS), when cohort members were 17 years old. This was the only study we found that measured both SDQ and WEMWBS for the same participants. In contrast to our own study, the MCS used the short (7-item) version of the well-being scale, the SWEMWBS. Selecting the 25% participants with the highest SDQ total difficulties score (SDQ ≥ 15, *n* = 2473) gave us an estimate of SD = 4.7 for the SWEMWBS. We decided to assume the slightly larger SD = 5 for the calculations below, for simplicity and to err on the conservative side. It is not clear what size of points difference on the SWEMWBS is clinically meaningful or subjectively important to children, although change scores as small as either 1 or 3 points have been cited as minimally important change on the individual level [100]. Raw effect sizes of 1, 2 and 3 points would correspond to standardised effect sizes of about 0.2, 0.4 and 0.6. Since there is a high correlation between the 7-item SWEMWBS and the 14-item WEMWBS, we assume that these results will scale appropriately to the longer version of the scale, which we will use in our study.

The aim of the power analysis was to determine the number of schools that need to be recruited to give us 90% power to detect a moderate effect of the intervention (δ = 0.4) for each of the SDQ Total Difficulties Score and the WEMWBS. To correct for multiple testing of our two primary outcomes, we set alpha at 0.025 for a two-sided test. We will select larger than average schools such that each school year will have around 240 pupils or more. With 25% of pupils meeting the entry threshold, and estimating that 50% agree to take part, we considered that we are likely to recruit 30 students per school year, and thus 60 students per school. The group intervention was assumed to be delivered in groups of 10. Thus, we expect to run approximately three intervention groups per school.

We decided on 1:1 randomisation between treatment and control participants. We assumed the within-group correlation to be ICC_group_ = 0.05 and the within-year correlation to be ICC_year_ = 0.02, which are both conservative: in similar trials, intraclass correlations are often smaller (smaller ICCs would result in higher power; [96]). We also assumed a within-student autocorrelation from baseline to post-intervention measures of *r*_participant_ = 0.5. This is again conservative: the median within-participant correlation in trials is estimated to be 0.59 [101] (a higher *r*_participant_ would result in higher power). Furthermore, we assumed that the outcomes would not be related to school, which is also conservative; within-school correlation would increase the power, as each school would act as its own control. Finally, our analysis assumed up to 20% of participants lost to follow-up in both the treatment and control groups.

Under these assumptions, 540 adolescents from nine schools will need to be recruited: 270 in the intervention arm, attending 27 treatment groups, and 270 in the control arm. This would give an estimated analysis sample (after loss to follow-up) of 432. The power to detect δ = 0.4 in one outcome at the 2.5% significance level is about 92.8%. There is some room in this design for unexpected recruitment difficulties, as the power would be 90.5% if only 25 treatment groups were run and 250 controls were recruited, which would happen, for example, if two schools were able to run only two intervention groups each, instead of three.

## Methods: data collection, management, and analysis

### Data collection methods

#### Data collection plan

To promote the data quality, all assessors are trained in measurement protocols and are provided with a manual outlining the procedure for collecting assessment data to enhance fidelity to the data collection protocol. Furthermore, all measures are delivered via a tablet, meaning participants do not report primary or secondary outcomes under observation, reducing the chances of biased responses. A log of any issues that occur during data collection will be recorded to monitor data quality throughout the duration of the project. All measures have previously been used with developmental samples and have demonstrated good reliability and validity in these samples.

#### Data management

Data from the cognitive-emotional training tasks will be collected via mobile applications designed to host the training tasks on tablets. Data from the emotion perception training task and emotion regulation training task will be collected via PsyTools, a software designed for hosting behavioural tasks and questionnaires. Data from the interoception task will be collected using a bespoke application that is uploaded to Firebase, a secure data storage server (https://firebase.google.com/docs/projects/learn-more?authuser=2&hl=en). Data from these tasks are stored on the host device and immediately uploaded to secure servers using encrypted transfer procedures. If there is no internet connection, data are encrypted and stored on the device until an internet connection has been established. No data from these tasks can be linked to individual participants from the servers nor the devices themselves. Anonymised data is transferred from the secure servers to UCL’s Data Safe Haven (https://www.ucl.ac.uk/isd/services/file-storage-sharing/data-safe-haven-dsh), a secure repository for storing, handling, and analysing data. Only the research team will have access to the data on UCL’s Data Safe Haven.

Data from the focus groups will be collected on an audio recording device that is only accessible to the research team. Audio recordings will then be uploaded to UCL’s Data Safe Haven for transcription. Once audio files have been transcribed, they will be securely destroyed.

### Data management

Data will be managed by an external company who provide data management services: Delosis. This company will be responsible for transferring data from the PsyTools app or extracting the data from the Firebase database. Our data management plan can be found in the Additional file 1: Appendix.

### Statistical methods

#### Primary outcome analysis

The primary analyses aim to estimate the effect of the intervention on two primary outcomes: psychopathology and mental wellbeing. Psychopathology will be measured as the self-reported SDQ Total Difficulties score. The other primary outcome, mental wellbeing, will be assessed by the summary score on the Edinburgh Mental Wellbeing Scale.

The primary outcome analysis will use a partially clustered mixed effects model [102], which takes into account that participants are clustered in treatment groups in the intervention arm, but not in the control arm, via a random effect for treatment groups. The model will adjust for baseline score of the outcome measure. We will additionally control for clustering of all participants within school years via a fixed effect, and for clustering within schools via either fixed or random effects (details will be specified in the statistical analysis plan). The evidence for a treatment effect will be evaluated via a two-sided *t*-test on the coefficient of the treatment indicator variable (intervention versus control group) from the mixed effects model, using a 2.5% level of significance. Analyses will be carried out in the R software (R Core Team 2021). The primary endpoint will be post-intervention (see Fig. 4).

#### Secondary analyses

Analogous partially clustered models will be estimated for.

- Each primary outcome at 1-year follow-up
- Each secondary outcome at both end of intervention and 1-year follow-up

#### Further analyses

**Fig. 6:**
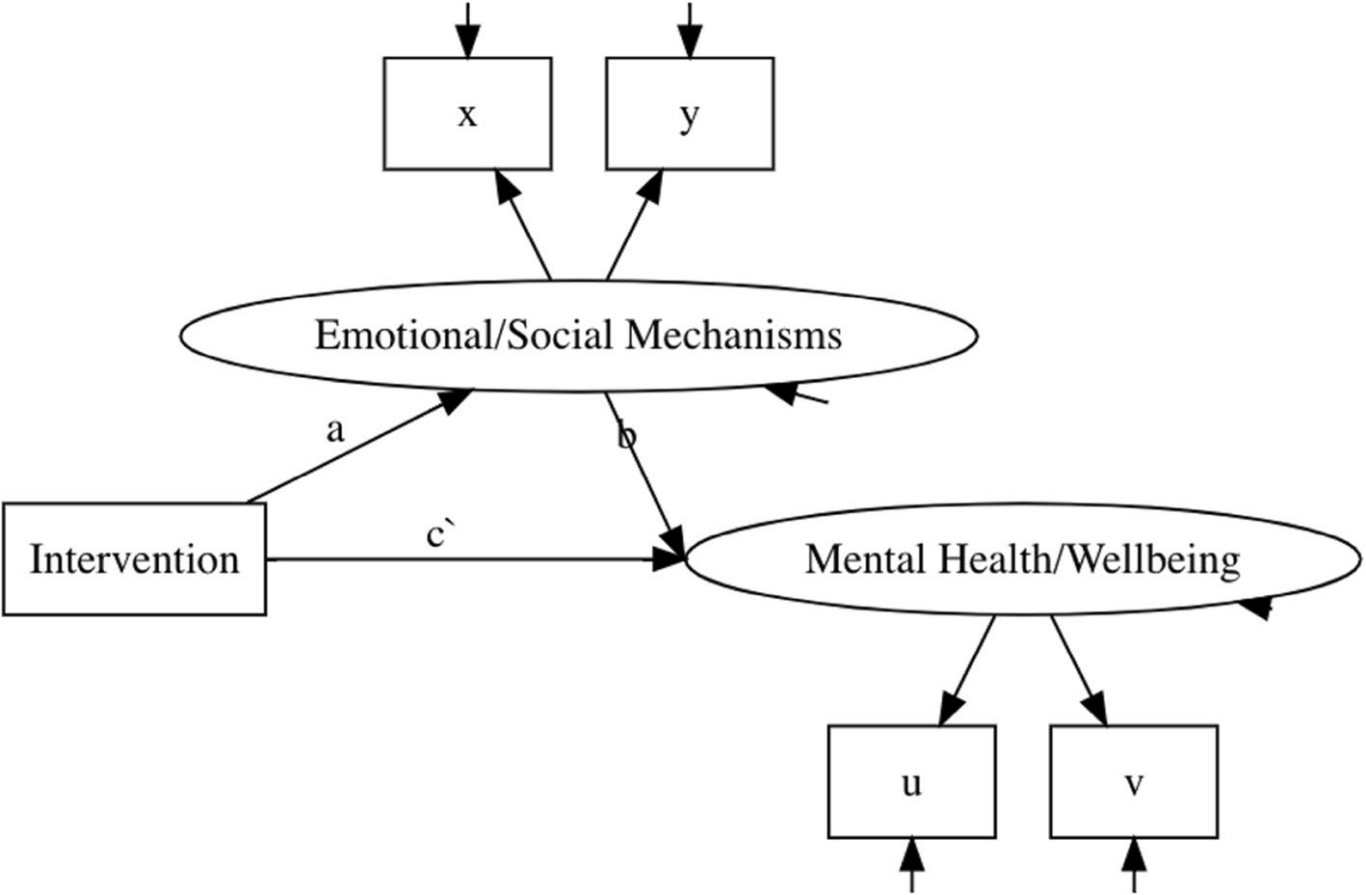
Plot illustrating the proposed SEM examining the indirect effects of treatment and mediators on outcomes. Note, the measurement model for the latent factors here show to items per factor (x, y and u, v), but in our final model there will be more items per factor. Clustering in treatment groups is not represented in this diagram, but will be accounted for in the modelling

Using structural equation modelling techniques, we will examine mediation of treatment effects using bootstrapped estimates of the indirect effect between treatment, mediator and outcome, as illustrated in Fig. 6 below. The mediation models will respect the clustered data structure and control for baseline outcome scores. A full analysis plan will be pre-registered on the Open Science Framework prior to the start of the analysis (see: https://osf.io/34jur/).

### Consent and assent procedures

All consent forms utilised in the current study will be sent to parents and adolescents by staff members at the participating schools. Consent forms will be provided in electronic or paper format, depending on the school’s typical procedure for acquiring parental consent. Electronic consent forms will be stored in secure servers provided by REDCap (https://projectredcap.org/) and physical copies will be stored in a secure filing cabinet only accessible to the study team.

### Data monitoring

Data security will undergo regular reviews by the research team to ensure it is compliant with our data management plan (see Additional file 1: Appendix). This data management plan will be reviewed annually to ensure no new risks are identified. The data will additionally be monitored by the data management provider.

### Ethics and risk management

All procedures involved in the study have been approved by UCL’s ethics committee (project number: 21815/001). We have designed the study materials to minimise the risk of harm to participants and have a strict safeguarding protocol in place. Those who wish to take part are provided with full, transparent information about the aims and content of the study to ensure that they can provide informed consent. Moreover, participants are reminded that they are free to withdraw from the study at any stage without providing an explanation. To ensure that there is no risk of harm to participants, we have utilised measures and task that have previously been used with this age group in previous research and are therefore appropriate for developmental populations.

We have also ensured that members of the research team are appropriately qualified to work with developmental populations. All members of the research team who work with adolescents or vulnerable adults will have a current Disclosure and Barring Service (DBS) certificate and will receive specific training about safeguarding. A risk-related triaging will be applied, such that urgent concerns are reported immediately to a clinically qualified member of the team (e.g. RL or PF), who can advise about the appropriate action and consult with the safeguarding lead at the Anne Freud Centre if necessary. Facilitators delivering the sessions will all be provided with bespoke training designed for the purposes of the course and delivered by the senior clinician on the team (RL). The training will be comprised of a minimum of 2 days of training with additional resources such as educational videos and a manual that facilitators can refer to. In addition, clinicians delivering the intervention are senior, experienced practitioners with expertise in the delivery of therapeutic interventions. These clinicians also have up to date training in identifying and referring high-risk participants, and any adolescents exhibiting symptoms of distress will be triaged according to the school’s policy regarding pupil wellbeing. Any adverse events or serious adverse events related to the trial will be stored confidentially by the study team and will be reported alongside the main trial outcomes. We define an adverse event as an incident that requires referral to a Child and Young Person Mental Health Support Team and/or GP (see Appendix III for a copy of the adverse event reporting form). 

### Trial oversight

The delivery of the pilot, feasibility, and main trial has been subject to the oversight of an advisory committee comprised of stakeholders who are independent to the project. Membership of this advisory committee includes academics, education practitioners, policy experts, parents, and young people. This committee meets quarterly to review and audit progress on the project, acting as both the trial steering committee and data monitoring committee. Should this advisory committee identify any concerns, whether these be to the study participants or the study protocol, they can make recommendations to halt any further progress on the project. Should the advisory group make a recommendation to halt the project, a complete review of the materials and protocol will be conducted by the research team.

### Research ethics approval

This pilot and feasibility studies and the main trial have all received approval from University College London’s ethics board (ref: 21815/001) and NHS Health Research Authority (IRAS project ID: 322531; REC reference: 23/NW/0145). All procedures are compliant with the British Psychological Society’s ethical guidance for research with human and non-human animal participants.

### Protocol amendments

Any amendments to the protocol will be submitted to UCL’s ethics committee for approval. Such amendments may include (but are not limited to) changes to the materials used, changes to the mode of delivery of the intervention, method of data collection or changes to the informed consent procedure. Changes to the study protocol that have limited or no impact on the risk of harm to participants or the project will not be submitted to the ethics committee but will be documented internally and published as a memorandum.

### Confidentiality

All data related to the study will be stored on secure, encrypted devices and databases. Any physical materials related to the intervention will be stored safely at the intervention site in a locked container. Physical materials used in the intervention are not research data and therefore these resources will not be seen by the research team. Any materials related to the evaluation of the pilot study (e.g. cognitive-emotion training task data, interview transcripts) will be stored securely on encrypted devices and only the research team will have access to these data.

### Declarations of interests

The authors do not have any interests to declare.

### Access to data

Anonymised data will be made publicly available via UCL’s Data Safe Haven as well as on the Open Science Framework. Participants who do not provide consent for their data to be shared publicly will still be recruited for the study. However, these data will be excluded from the publicly available dataset. For quantitative data, participants will be provided with a pseudonymised identifier, though no personal information will be made publicly available. For qualitative data, transcripts will be uploaded with any personally identifiable details redacted from the transcripts. These data sharing policies are detailed in the information and consent forms provided to participants and their guardians.

### Ancillary and post-trial care

Participants will be provided with a debrief form that includes contact details for charities that support young people with their mental health at the end of the study. This debrief form will also contain contact details for the researchers should participants have any questions or concerns regarding their participation. The debrief form will also include information about how participants may retract their data should they wish to withdraw from the study post hoc. The research team will maintain contact with staff members at the host school in case further post-trial care is required.

### Publication and dissemination plan

Published scientific protocol, intervention manual, implementation toolkit, dissemination co-produced by young people (including two films, podcasts, a webinar), scientific papers (intervention development, process evaluation, intervention impact, mediating mechanisms, reciprocal development of emotion processing and social relationships and their impact on mental health), briefing documents, conference presentations, and curated dataset for scientific usage are intended outputs from the trial. Authorship will be determined using the Contributor Roles Taxonomy (CRediT).

## Trial status

*Protocol version number*: 2

*Recruitment start date*: 09/03/2023.

*Approximate recruitment completion:* 31/03/2024.

### Supplementary Information

**Additional file 1.** **Appendices.** I: R script to determine school randomisation.

**Additional file 2.** SPIRIT Checklist for Trials.

**Additional file 3.** Appendix 3.

## Data Availability

Anonymised data will be made publicly available via UCL’s Data Safe Haven as well as on the Open Science Framework. Participants who do not provide consent for their data to be shared publicly will still be recruited for the study. However, these data will be excluded from the publicly available dataset.
