# Supplementary material for: Trial protocol for the Building Resilience through Socio-Emotional Training (ReSET) programme: a cluster randomised controlled trial of a new transdiagnostic preventative intervention for adolescents
Source: Trials. 2024 Feb 23;25:143. doi: 10.1186/s13063-024-07931-2 (PMC10885387; doi:10.1186/s13063-024-07931-2)
Supplement: Supplementary file 1 — Additional file 1. Appendices. I: R script to determine school randomisation. [file 13063_2024_7931_MOESM1_ESM.docx]

**Appendices**

**I: R script to determines school randomisation**

#RE-SET randomization programme

#Peter Martin, University College London

#June 2022

#Define block elements

EL = c("Cohort1-INT", "Cohort2-INT")

#Define number of blocks

M = 6

#Function to select 1 block

block = function(i) {

block = sample(EL, 2, replace = FALSE, prob = c(.5, .5))

return(block)

}

#function to select M blocks

blocklist = function(EL, M) {

blocktab = sapply(1:M, block)

firsts = (1:M)*2-1

seconds = (1:M)*2

allocation_list = rep(NA, 2*M)

for (j in 1:M) {

allocation_list[firsts[j]] = blocktab[1,j]

allocation_list[seconds[j]] = blocktab[2,j]

}

return(allocation_list)

}

#Set random seed

set.seed(123456) #Change seed for random allocation

#Create random list

allocation_list = blocklist(EL, M)

#Processing

school_ids = 1:(2*M)

allocations = data.frame(school = school_ids, allocation = allocation_list)

allocations

write.csv(allocations, "RESET randomized list [add date] (test).csv")

**II: Sleep Phenotype Questionnaire**

1. During the past month, when have you usually gone to bed at night **in the week**?

Please give you answer as it would appear on a 24-hour clock (e.g., 8am would be 08:00 and 8pm would be 20:00)

1. During the past month, when have you usually gone to bed at night **on the weekend**?

Please give you answer as it would appear on a 24-hour clock (e.g., 8am would be 08:00 and 8pm would be 20:00)

1. During the past month, when have you usually gotten up in the morning **in the week**?

Please give you answer as it would appear on a 24-hour clock (e.g., 8am would be 08:00 and 8pm would be 20:00)

1. During the past month, when have you usually gotten up in the morning **on the weekend**?

Please give you answer as it would appear on a 24-hour clock (e.g., 8am would be 08:00 and 8pm would be 20:00)

1. During the past month, how many hours of actual sleep did you get at night? (This may be different than the number of hours you spend in bed)
2. During the past month, how would you rate your sleep quality overall?

| Very bad | Fairly bad | Fairly good | Very good |
| --- | --- | --- | --- |
| 1 | 2 | 3 | 4 |

1. During the past month how often have you had difficulty falling asleep, staying asleep or have had a problem with waking too early?

| 1 time a week | 2 times a week | 3 times a week | 4 times a week | 5 times a week | 6 times a week | 7 times a week |
| --- | --- | --- | --- | --- | --- | --- |

1. If you have reported that you have had difficulties sleeping to what extent has this led to daytime impairment?

| Not at all | A little | Somewhat | Much | Very much |
| --- | --- | --- | --- | --- |
| 1 | 2 | 3 | 4 | 5 |

1. One hears about ‘morning’ and ‘evening’ types of people. Which ONE of these types do you consider yourself to be?

| Definitely a ‘morning’ type | Rather a ‘morning’ than an ‘evening’ type | Rather more an ‘evening’ than ‘morning type | Definitely an ‘evening’ type |
| --- | --- | --- | --- |
| 1 | 2 | 3 | 4 |

**IV: Data Management Plan**

| **0. Proposal name** | |
| --- | --- |
| Developing a school-based, transdiagnostic, preventative intervention for adolescent mental health | |
| **1. Description of the data** | |
| **Type of study:** Mixed methods study to develop a new hybrid preventative intervention, focusing on emotion processing and social relationship mechanisms, to promote mental health and wellbeing in high risk adolescents. **Types of data:** *Quantitative data* from self-report and teacher report questionnaires, experimental psychology tasks collected via tablets and smartphone, and social network data collected among peers in the same year group; *qualitative data* from focus groups. **Format and scale of the data:** *S*elf-report and teacher report questionnaires, experimental psychology tasks and peer nominations will be obtained for 540 adolescents (270 in intervention and 270 in non-intervention arm of the study) on three occasions. Basic screening information on mental health will be collected from approximately 4320 pupils from which the study sample is drawn. Data will be stored securely in excel, text file, comma-separate value (CSV), SPSS, or R (Rds) format. To ensure optimum accessibility to relevant users we will make anonymized data (where participants have consented to data sharing) open access on the UCL’s Research Data Repository (<https://rdr.ucl.ac.uk>), which will be accompanied by a data dictionary and statistical summary report. All qualitative focus group interviews will be recorded using secure digital audio recorders and downloaded to encrypted study laptops prior to depositing on UCL’s Data Safe Haven. Data files will be stored in mp3 format. Transcribed audio recordings will be edited to remove all identifiers and stored as word documents on UCL’s Data Safe Haven. | |
| **2. Data collection / generation** | |
| **Methodologies for data collection / generation:** *Quantitative:* Self-report questionnaires, teacher-report questionnaires, tablet and phone administered tasks and social network data (peer nominations) are collected during testing/intervention sessions in the school setting via tablets (and in the case of interoception data, via smartphones (study owned or own) and wearables (study owned)) via Psytools platform or a specialist app (in the case of interoception data). *Qualitative:* Up to 15 focus group interviews will be conducted with approximately 6-8 participants/group. All interviews will be digitally audio-recorded and transcribed using a secure professional transcription service. Once transcriptions are complete all audio recordings will be deleted. Transcribed interviews will be anonymised by removing by hand any identifying information (names, places etc), and then subjected to framework analysis. **Data quality and standards:** *Quantitative:* Research staff will be trained in the administration of the research instruments and specific Standard Operating Procedures (SOPs) will be developed for each distinct element of data collection. Prior to data collection activity, staff will be trained in data protection procedures and regulations, Good Clinical Practice and information governance as well as all the study-specific SOPs. Video recordings will be made of the desired testing protocol to maintain consistency between assessors and across time. All questionnaire data will be administered via tablets to reduce risk of data entry errors; the interface will ensure data completeness and contain standard validation rules to prevent inadmissible entries (e.g., for dates, valid numeric ranges). All assessment processes will be piloted prior to formal data collection begins. *Qualitative:* Staff who are gathering data as part of focus groups will receive general training in interviewing skills, and specific training in relation to the interview schedule. SOPs will be developed for focus group interviews (including transcription guidelines), including how to manage confidentiality, disclosures related to risk and safeguarding, as well as responding to distress. Recordings of interviews will be reviewed by the qualitative research lead as part of regular supervision, to ensure quality of facilitation; and a selection of transcripts will be peer-reviewed to check accuracy. | |
| **3. Data management, documentation and curation** | |
| The proposed study involves quantitative questionnaire and experimental task data, cognitive training data, as well as qualitative data from focus groups. All questionnaire, experimental task and training data will be collected on computers or tablet/phone applications at school. These data will be stored in tablets used for data collection, immediately sent for a secure server back-up (via secure link) upon collection, and later moved to the computer used for data analysis, as well as being backed up in UCL’s Data Safe Haven. Data format depends on the software used, but data will be ultimately stored in excel, text file, comma-separate value (CSV), SPSS, or R formats. For analysis and publication of data, data for each task will be combined across all participants into one table, by adding participant-specific information (participant code, participant group, age, gender etc). All relevant data are stored in a single file, with critical information easily identifiable through column headings. To ensure optimum accessibility to relevant users we will make data (where participants have consented to data sharing) open access on the UCL’s Research Data Repository (<https://rdr.ucl.ac.uk>) in formats that are easily accessible and allow for flexible re-analysis of datasets by any interested person. All qualitative focus group interviews will be recorded using encrypted digital audio recorders and immediately transferred to password protected and encrypted laptops for transit back to the university. No audio recordings will be left on digital audio recorders after a testing session within a school for reasons of data security. Audio recordings will be transferred to UCL’s Data Safe Haven for storage in mp3 format, and sent securely from the Data Safe Haven to an NHS compliant secure transcription service for transcribing. Transcribed audio recordings will be edited to remove all identifiers and stored as word documents on UCL’s Data Safe Haven. In accordance with research council recommendations, data will be stored for 20 years post completion of the study. | |
| **4. Data security and confidentiality of potentially disclosive information** | |
| **Formal information/data security standards:** UCL data security standard information is provided in section 7. Participant names will be collected on consent/assent forms and in relation to social network data, which will be securely stored. An additional data key document that connects participant names to study identity codes will be stored in the UCL Data Safe Haven (ISO/IEC 27001:2013 Standard; Certificate/licence number IS 612909) and will enable removal of data upon participant request. **Main risks to data security:** Data are stored on servers and hardware managed by the UCL IT Services. Data management includes physical hardware security (password protection, encryption, authentication), data backup, disaster recovery processes, controlled access and secure disposal within an agreed time-frame. Access to data is strictly controlled. Any identifiable data are stored separately and linked to rest of the research data using a study identifier. All the processes of the study comply with the Data Protection Act and the General Data Protection Regulations (GDPR). The data are stored in two open source formats (CSV and XML) in UCL Data Safe Haven, specifically designed for secure long term storage of sensitive data. The qualitative interviews will be recorded using a securely encrypted digital audio recorder. The encrypted recordings will be transferred to the Data Safe Haven following each qualitative focus group session, and transcription will be done by an NHS data governance-compliant professional transcription service. Fully informed consent will be sought from focus group interview participants. Because qualitative focus group interviews produce data that can contain personal and identifiable information, interview participants will be assigned with a code, and their details, along with the digital recordings will be stored with strict protocols in accordance with the Data Protection Act 2018 and GDPR (locked filing cabinets, password protected University computes with encrypted data files, Data Safe Haven). Only PIs and project researchers working on data will have access to personal and identifiable information stored on the Data Safe Haven. Any information (such as quotations) extracted from the qualitative interviews will be presented in a way that guarantees confidentiality and anonymity. All data holdings are subject to UCL measures designed to prevent unauthorised processing of data, accidental data loss, or destruction of data. Quantitative data will be returned from password protected tablets to password protected University desktop computers and data stored on University secure server. Encrypted audio files will be transferred to a password protected University desktop computer immediately after each focus group, and stored on UCL’s Data Safe Haven. Personal and identifiable information will only be available to the PIs and project researchers and will be stored in Data Safe Haven. If a participant later withdraws their consent, their data will be destroyed securely. | |
| **5. Data sharing and access** | |
| **Suitability for sharing:** Once identifying information has been removed, numeric data will be suitable for sharing and will be shared via UCL’s research data repository (https://www.ucl.ac.uk/isd/services/research-it/research-data-repository). Prior to storing data, it will be de-identified and pseudonymised to avoid participant identification. Participants and their caregivers will be provided clear information about our data management and access protocols as part of the consent/assent process. As part of this process they will sign a Data Access Agreement that outlines the acceptable uses and behaviours relating to the data.  **Discovery by potential users of the research data:** Potential new users will be able to find out about the data via UCL research data repository (see section 7- Other) and made available upon application to suitably qualified researchers under a data usage agreement. As the study tests a preventative intervention the protocol will be registered on the ISRCTN trials registry, which will include links to the study website and the data repository. The UCL research data repository will include information on how the data can be accessed, how participants were recruited, summary information about the variables and information about data quality checks. Data deposited with the UCL research data repository is issued with a unique Digital Object Identifier (DOI) which can be cited in publications, increasing visibility of this resource. Governance of access: **The study team’s exclusive use of the data:** The team that designed the study has the expertise to execute the analyses answering the outlined research questions will have a 12 month period of exclusive use of the data from the end of the project before sharing it with the wider research community. This will also ensure that early career researchers linked to the study effort are appropriately supported in career investments that they make for the study. **Restrictions or delays to sharing, with planned actions to limit such restrictions:** All data sharing will be discussed with the study participants (parents and young people) and we will seek specific consent or assent to anonymised research data sharing outside the study team. In this context it will be clearly explained how the data are stored, what checks are performed to ensure that legitimate persons have access to data and what purposes would data sharing be appropriate for. Procedures to de-identify data will be explained thoroughly to reassure the participants that they could not be identified via data shared via a data repository. **Regulation of responsibilities of users:** External researchers that seek to use data from this study will be bound by data sharing and confidentiality agreements as outlined in section 7. | |
| **6. Responsibilities** | |
| Alex Potts is the Data Protection Officer is responsible for data security at UCL. In addition to the PIs the study manager will be responsible for study-wide data management, and, together with the team, quality assurance of data. | |
| **7. Relevant institutional, departmental or study policies on data sharing and data security** | |
| **Policy** | **URL or Reference** |
| Data Sharing Policies | <https://www.ucl.ac.uk/isd/sites/isd/files/ucl_research_data_policy_v6.pdf>  <https://www.ucl.ac.uk/library/research-support/research-data-management/best-practices/how-guides/sharing-data> |
| Data Management Policy & Procedures | <https://www.ucl.ac.uk/library/research-support/research-data-management>  <https://www.ucl.ac.uk/isd/sites/isd/files/ucl_research_data_policy_v6.pdf> |
| Data Security Policy | <https://www.ucl.ac.uk/information-security/sites/information-security/files/policy.pdf>  <https://www.ucl.ac.uk/isd/services/file-storage-sharing> |
| Other | <https://www.ucl.ac.uk/library/research-support/research-data-management/ucl-research-data-repository> |
| **8. Author of this Data Management Plan (Name)** and, if different to that of the Principal Investigator, their **telephone & email contact details** | |
| Essi Viding (Co-PI)/Pasco Fearon (Co-PI) | |

|  |  | **Reporting Item** | **Page and Line Number** | **Reason if not applicable** |
| --- | --- | --- | --- | --- |
| **Administrative information** | | | | |
| Title | [#1](https://www.goodreports.org/reporting-checklists/spirit/info/#1) | Descriptive title identifying the study design, population, interventions, and, if applicable, trial acronym | 1; 4-5 |  |
| Trial registration | [#2a](https://www.goodreports.org/reporting-checklists/spirit/info/#2a) | Trial identifier and registry name. If not yet registered, name of intended registry | 3; 2 |  |
| Trial registration: data set | [#2b](https://www.goodreports.org/reporting-checklists/spirit/info/#2b) | All items from the World Health Organization Trial Registration Data Set | 3-5; |  |
| Protocol version | [#3](https://www.goodreports.org/reporting-checklists/spirit/info/#3) | Date and version identifier | 5; 23 |  |
| Funding | [#4](https://www.goodreports.org/reporting-checklists/spirit/info/#4) | Sources and types of financial, material, and other support | 2; 6 |  |
| Roles and responsibilities: contributorship | [#5a](https://www.goodreports.org/reporting-checklists/spirit/info/#5a) | Names, affiliations, and roles of protocol contributors | 2-3; |  |
| Roles and responsibilities: sponsor contact information | [#5b](https://www.goodreports.org/reporting-checklists/spirit/info/#5b) | Name and contact information for the trial sponsor | 3; 7-9 |  |
| Roles and responsibilities: sponsor and funder | [#5c](https://www.goodreports.org/reporting-checklists/spirit/info/#5c) | Role of study sponsor and funders, if any, in study design; collection, management, analysis, and interpretation of data; writing of the report; and the decision to submit the report for publication, including whether they will have ultimate authority over any of these activities | 2; 12-14 |  |
| Roles and responsibilities: committees | [#5d](https://www.goodreports.org/reporting-checklists/spirit/info/#5d) | Composition, roles, and responsibilities of the coordinating centre, steering committee, endpoint adjudication committee, data management team, and other individuals or groups overseeing the trial, if applicable (see Item 21a for data monitoring committee) | 46; 14-23 |  |
| **Introduction** |  |  | 8-15; all lines |  |
| Background and rationale | [#6a](https://www.goodreports.org/reporting-checklists/spirit/info/#6a) | Description of research question and justification for undertaking the trial, including summary of relevant studies (published and unpublished) examining benefits and harms for each intervention | 8-13; all lines |  |
| Background and rationale: choice of comparators | [#6b](https://www.goodreports.org/reporting-checklists/spirit/info/#6b) | Explanation for choice of comparators | 10; 5-25 & 11; 1-25 and 13; 9-18 |  |
| Objectives | [#7](https://www.goodreports.org/reporting-checklists/spirit/info/#7) | Specific objectives or hypotheses | 14; 6-16 |  |
| Trial design | [#8](https://www.goodreports.org/reporting-checklists/spirit/info/#8) | Description of trial design including type of trial (eg, parallel group, crossover, factorial, single group), allocation ratio, and framework (eg, superiority, equivalence, non-inferiority, exploratory) | 14; 17-23 and 15; 1-5 |  |
| **Methods: Participants, interventions, and outcomes** | | | | |
| Study setting | [#9](https://www.goodreports.org/reporting-checklists/spirit/info/#9) | Description of study settings (eg, community clinic, academic hospital) and list of countries where data will be collected. Reference to where list of study sites can be obtained | 15; 8-11 |  |
| Eligibility criteria | [#10](https://www.goodreports.org/reporting-checklists/spirit/info/#10) | Inclusion and exclusion criteria for participants. If applicable, eligibility criteria for study centres and individuals who will perform the interventions (eg, surgeons, psychotherapists) | 15; 12-24 & 16; 1-14 |  |
| Interventions: description | [#11a](https://www.goodreports.org/reporting-checklists/spirit/info/#11a) | Interventions for each group with sufficient detail to allow replication, including how and when they will be administered | 16; 15-23 and 17; 1-24 and 18; 1-25 and 19; 1-19; and 20; 1-25 and 21; 1-23 and 22; 1-18 and 23; 1-15 and 24; 1-24 |  |
| Interventions: modifications | [#11b](https://www.goodreports.org/reporting-checklists/spirit/info/#11b) | Criteria for discontinuing or modifying allocated interventions for a given trial participant (eg, drug dose change in response to harms, participant request, or improving / worsening disease) | 17; 13-21 |  |
| Interventions: adherance | [#11c](https://www.goodreports.org/reporting-checklists/spirit/info/#11c) | Strategies to improve adherence to intervention protocols, and any procedures for monitoring adherence (eg, drug tablet return; laboratory tests) | 25; 1-25 |  |
| Interventions: concomitant care | [#11d](https://www.goodreports.org/reporting-checklists/spirit/info/#11d) | Relevant concomitant care and interventions that are permitted or prohibited during the trial | 15; 23-24 and 16; 1-3 |  |
| Outcomes | [#12](https://www.goodreports.org/reporting-checklists/spirit/info/#12) | Primary, secondary, and other outcomes, including the specific measurement variable (eg, systolic blood pressure), analysis metric (eg, change from baseline, final value, time to event), method of aggregation (eg, median, proportion), and time point for each outcome. Explanation of the clinical relevance of chosen efficacy and harm outcomes is strongly recommended | 26; 9-24 and 27; 1-23 and 28; 1-25 and 29; 1-24 and 30; 1-23 and 31; 1-24 and 32; 1-19 |  |
| Participant timeline | [#13](https://www.goodreports.org/reporting-checklists/spirit/info/#13) | Time schedule of enrolment, interventions (including any run-ins and washouts), assessments, and visits for participants. A schematic diagram is highly recommended (see Figure) | 37; 10-24 and 38; 1-7 see also Figure 6 |  |
| Sample size | [#14](https://www.goodreports.org/reporting-checklists/spirit/info/#14) | Estimated number of participants needed to achieve study objectives and how it was determined, including clinical and statistical assumptions supporting any sample size calculations | 40; 1-24 and 41; 1-24 and 42; 1-24 and 43; 1-2 |  |
| Recruitment | [#15](https://www.goodreports.org/reporting-checklists/spirit/info/#15) | Strategies for achieving adequate participant enrolment to reach target sample size | 37; 10-21 |  |
| **Methods: Assignment of interventions (for controlled trials)** | | | | |
| Allocation: sequence generation | [#16a](https://www.goodreports.org/reporting-checklists/spirit/info/#16a) | Method of generating the allocation sequence (eg, computer-generated random numbers), and list of any factors for stratification. To reduce predictability of a random sequence, details of any planned restriction (eg, blocking) should be provided in a separate document that is unavailable to those who enrol participants or assign interventions | 36; 7-20 |  |
| Allocation concealment mechanism | [#16b](https://www.goodreports.org/reporting-checklists/spirit/info/#16b) | Mechanism of implementing the allocation sequence (eg, central telephone; sequentially numbered, opaque, sealed envelopes), describing any steps to conceal the sequence until interventions are assigned | 36; 14-20 and 37; 1-8 |  |
| Allocation: implementation | [#16c](https://www.goodreports.org/reporting-checklists/spirit/info/#16c) | Who will generate the allocation sequence, who will enrol participants, and who will assign participants to interventions | 36; 14-20 |  |
| Blinding (masking) | [#17a](https://www.goodreports.org/reporting-checklists/spirit/info/#17a) | Who will be blinded after assignment to interventions (eg, trial participants, care providers, outcome assessors, data analysts), and how | 37; 1-8 |  |
| Blinding (masking): emergency unblinding | [#17b](https://www.goodreports.org/reporting-checklists/spirit/info/#17b) | If blinded, circumstances under which unblinding is permissible, and procedure for revealing a participant’s allocated intervention during the trial | 37; 1-8 |  |
| **Methods: Data collection, management, and analysis** | | | | |
| Data collection plan | [#18a](https://www.goodreports.org/reporting-checklists/spirit/info/#18a) | Plans for assessment and collection of outcome, baseline, and other trial data, including any related processes to promote data quality (eg, duplicate measurements, training of assessors) and a description of study instruments (eg, questionnaires, laboratory tests) along with their reliability and validity, if known. Reference to where data collection forms can be found, if not in the protocol | 43; 5-24 and 44; 1-8 |  |
| Data collection plan: retention | [#18b](https://www.goodreports.org/reporting-checklists/spirit/info/#18b) | Plans to promote participant retention and complete follow-up, including list of any outcome data to be collected for participants who discontinue or deviate from intervention protocols | 35; 3-9 |  |
| Data management | [#19](https://www.goodreports.org/reporting-checklists/spirit/info/#19) | Plans for data entry, coding, security, and storage, including any related processes to promote data quality (eg, double data entry; range checks for data values). Reference to where details of data management procedures can be found, if not in the protocol | 44; 9-13 and Appendix IV |  |
| Statistics: outcomes | [#20a](https://www.goodreports.org/reporting-checklists/spirit/info/#20a) | Statistical methods for analysing primary and secondary outcomes. Reference to where other details of the statistical analysis plan can be found, if not in the protocol | 44; 15-23 and 45; 1-6 |  |
| Statistics: additional analyses | [#20b](https://www.goodreports.org/reporting-checklists/spirit/info/#20b) | Methods for any additional analyses (eg, subgroup and adjusted analyses) | 45; 7-18 and Figure 7 |  |
| Statistics: analysis population and missing data | [#20c](https://www.goodreports.org/reporting-checklists/spirit/info/#20c) | Definition of analysis population relating to protocol non-adherence (eg, as randomised analysis), and any statistical methods to handle missing data (eg, multiple imputation) | 44; 15-23 and 45; 1-6 |  |
| **Methods: Monitoring** | | | | |
| Data monitoring: formal committee | [#21a](https://www.goodreports.org/reporting-checklists/spirit/info/#21a) | Composition of data monitoring committee (DMC); summary of its role and reporting structure; statement of whether it is independent from the sponsor and competing interests; and reference to where further details about its charter can be found, if not in the protocol. Alternatively, an explanation of why a DMC is not needed | 48; 4-13 |  |
| Data monitoring: interim analysis | [#21b](https://www.goodreports.org/reporting-checklists/spirit/info/#21b) | Description of any interim analyses and stopping guidelines, including who will have access to these interim results and make the final decision to terminate the trial | 49; 13-21 |  |
| Harms | [#22](https://www.goodreports.org/reporting-checklists/spirit/info/#22) | Plans for collecting, assessing, reporting, and managing solicited and spontaneously reported adverse events and other unintended effects of trial interventions or trial conduct | 47; 3-24 and 48; 1-3 and 50; 1-7 |  |
| Auditing | [#23](https://www.goodreports.org/reporting-checklists/spirit/info/#23) | Frequency and procedures for auditing trial conduct, if any, and whether the process will be independent from investigators and the sponsor | 48; 4-13 |  |
| **Ethics and dissemination** | | | | |
| Research ethics approval | [#24](https://www.goodreports.org/reporting-checklists/spirit/info/#24) | Plans for seeking research ethics committee / institutional review board (REC / IRB) approval | 5; 4-6 and 48; 14-19 |  |
| Protocol amendments | [#25](https://www.goodreports.org/reporting-checklists/spirit/info/#25) | Plans for communicating important protocol modifications (eg, changes to eligibility criteria, outcomes, analyses) to relevant parties (eg, investigators, REC / IRBs, trial participants, trial registries, journals, regulators) | 48; 20-24 and 49; 1-2 |  |
| Consent or assent | [#26a](https://www.goodreports.org/reporting-checklists/spirit/info/#26a) | Who will obtain informed consent or assent from potential trial participants or authorised surrogates, and how (see Item 32) | 46; 7-13 |  |
| Consent or assent: ancillary studies | [#26b](https://www.goodreports.org/reporting-checklists/spirit/info/#26b) | Additional consent provisions for collection and use of participant data and biological specimens in ancillary studies, if applicable |  | n/a This trial does not involve collecting biological specimens for storage. |
| Confidentiality | [#27](https://www.goodreports.org/reporting-checklists/spirit/info/#27) | How personal information about potential and enrolled participants will be collected, shared, and maintained in order to protect confidentiality before, during, and after the trial | 49; 3-9 |  |
| Declaration of interests | [#28](https://www.goodreports.org/reporting-checklists/spirit/info/#28) | Financial and other competing interests for principal investigators for the overall trial and each study site | 49; 11-12 |  |
| Data access | [#29](https://www.goodreports.org/reporting-checklists/spirit/info/#29) | Statement of who will have access to the final trial dataset, and disclosure of contractual agreements that limit such access for investigators | 49; 13-21 |  |
| Ancillary and post trial care | [#30](https://www.goodreports.org/reporting-checklists/spirit/info/#30) | Provisions, if any, for ancillary and post-trial care, and for compensation to those who suffer harm from trial participation | 47; 9-16 |  |
| Dissemination policy: trial results | [#31a](https://www.goodreports.org/reporting-checklists/spirit/info/#31a) | Plans for investigators and sponsor to communicate trial results to participants, healthcare professionals, the public, and other relevant groups (eg, via publication, reporting in results databases, or other data sharing arrangements), including any publication restrictions | 50; 1-7 |  |
| Dissemination policy: authorship | [#31b](https://www.goodreports.org/reporting-checklists/spirit/info/#31b) | Authorship eligibility guidelines and any intended use of professional writers | 50; 9-15 |  |
| Dissemination policy: reproducible research | [#31c](https://www.goodreports.org/reporting-checklists/spirit/info/#31c) | Plans, if any, for granting public access to the full protocol, participant-level dataset, and statistical code | 49; 14-15 |  |
| **Appendices** | | | | |
| Informed consent materials | [#32](https://www.goodreports.org/reporting-checklists/spirit/info/#32) | Model consent form and other related documentation given to participants and authorised surrogates |  | n/a attached as a separate document |
| Biological specimens | [#33](https://www.goodreports.org/reporting-checklists/spirit/info/#33) | Plans for collection, laboratory evaluation, and storage of biological specimens for genetic or molecular analysis in the current trial and for future use in ancillary studies, if applicable |  | n/a This trial does not involve collecting biological specimens for storage. |

I
