# Supplementary material for: Trial protocol for the Building Resilience through Socio-Emotional Training (ReSET) programme: a cluster randomised controlled trial of a new transdiagnostic preventative intervention for adolescents
Source: Trials. 2024 Feb 23;25:143. doi: 10.1186/s13063-024-07931-2 (PMC10885387; doi:10.1186/s13063-024-07931-2)
Supplement: Supplementary file 3 — Additional file 3. Appendix 3. [file 13063_2024_7931_MOESM3_ESM.docx]

**Adverse Event report form**

An adverse event report form should be completed for all **adverse events (AE)** which take place to ensure the event and the action that was taken by the research team is documented and available to for review if required. If the nominated senior clinician advises no action should be taken, an AE report form should still be completed to document this decision.

The local researcher or research assistant who received the report from the participant should complete the form. All sections of the form should be completed. If the requested information is not known, please indicate the information is “not known to the research team” in the table below. The name of the research participant or identifiable information (initials, DOB, address) should not be included anywhere on the form. The nominated senior clinician should review and approve the form before it is finalised. The completed form should be emailed to the central and local trial coordinators so they can update the central and local trial master files. The local chief investigator and nominated senior clinician should be cc’d into all email correspondence.

| **SECTION 1: DESCRIPTION OF THE EVENT** | |
| --- | --- |
| **Research site** |  |
| **Safeguarding concern identified?**  *Please select one option* | YES  NO |
| **Unique participant research ID** |  |
| **Date the event took place** |  |
| **Description of where the event took place** |  |
| **Description of who was present when the event was reported to the Local researcher or research assistant** |  |
| **Description and further information about the event** |  |
| **Additional information** |  |

| **SECTION 2: REPORTING THE EVENT** | |
| --- | --- |
| **Name and job role of the person(s) that the event was initially reported to** |  |
| **Date and local time the named person(s) above was informed of the event** |  |
| **Method(s) by which the named person(s) above was informed of the event** | *Please tick all the apply*  Email  Telephone  Other,  *If other please state ______________________* |
| **Having informed the named person above of the event described in Section 1, has any further action been advised?** | *Please select one option*  Yes, the event was classified by the nominated senior clinician as an AE, and therefore further action advised.  No, the event was not classified as an AE by the senior clinician and therefore no further action required.  **If the event was classified by the nominated senior clinician as an AE please complete section 3 below to describe the further action taken.** |

| **SECTION 3: FURTHER ACTION TAKEN** | |
| --- | --- |
| **Description of further action taken**  *Please include the following information:*   - *Dates and times of each communication* - *Names and job titles of professionals* - *Actions and decisions made* |  |

| **SECTION 4: DECLARATION** | |
| --- | --- |
| ***Details of the local researcher or research assistant who received the report; reported the event and completed the Adverse Event form.*** | |
| **Name** |  |
| **Job role** |  |
| **Organisation** |  |
| **Signature** |  |
| **Date** |  |
| ***Details of the nominated senior clinician who classified the severity of the event.*** | |
| **Name** |  |
| **Job role** |  |
| **Organisation** |  |
| **Signature** | I declare that the information I have provided in this form is, to the best of my knowledge, true and accurate. |
| **Date** |  |
